# Human umbilical cord mesenchymal stem cell-derived nanovesicles ameliorate acute lung injury by hsa-let-7g-5p inhibition of NF-κB/NLRP3 pathway

**DOI:** 10.20517/evcna.2025.170

**Published:** 2026-04-02

**Authors:** Yilin Huang, Yan Zeng, Ailin Wu, Yang Chen, Yuanhao Zhou, Youni Zhang, Hai Zou, Weijiao Fan, Xiaoyi Chen, Jinyang Chen, Jie Wang, Xianghong Yang, Xiaoru Chang, Xiaozhou Mou, Yuexing Tu

**Affiliations:** ^1^Center for Rehabilitation Medicine, Rehabilitation & Sports Medicine Research Institute of Zhejiang Province, Department of Rehabilitation Medicine, Translational Medicine Center, Zhejiang Provincial People’s Hospital, Hangzhou Medical College, Hangzhou 310014, Zhejiang, China.; ^2^College of Pharmacy, Hangzhou Medical College, Hangzhou 310059, Zhejiang, China.; ^3^Department of Pharmacy, Hubei Hospital of Integrated Chinese & Western Medicine, Wuhan 430015, Hubei, China.; ^4^Clinical Laboratory Department, Tiantai People’s Hospital of Zhejiang Province (Tiantai Branch of Zhejiang Provincial People’s Hospital), Hangzhou Medical College, Taizhou 317200, Zhejiang, China.; ^5^Department of Emergency and Critical Care Medicine, Shanghai Pudong New Area People’s Hospital, Shanghai 200120, China.; ^6^Weway (Hangzhou) Biotechnology Co., Ltd., Hangzhou 310052, Zhejiang, China.; ^7^EVital Bio (Hangzhou) Co., Ltd., Hangzhou 310056, Zhejiang, China.; ^8^Department of Critical Care Medicine, Tongde Hospital of Zhejiang Province, Hangzhou 310012, Zhejiang, China.; ^#^Authors contributed equally.

**Keywords:** Umbilical cord mesenchymal stem cell-derived nanovesicles, acute lung injury, inflammation, microRNA, NF-κB/NLRP3 inflammasome pathway

## Abstract

**Aim:** Acute lung injury (ALI), marked by vigorous inflammatory reactions and elevated incidence, is a grave clinical issue for which efficacious pharmaceutical interventions are still lacking. This research delves into the anti-inflammatory actions and the associated pathways of nanovesicles originating from human umbilical cord mesenchymal stem cells (UCMSC-NVs) within a model of ALI induced by lipopolysaccharide (LPS).

**Methods:** UCMSC-NVs were prepared via serial extrusion and characterized using transmission electron microscopy and dynamic light scattering. Their effects on pulmonary inflammation and injury were evaluated in an LPS-induced ALI mouse model. Anti-inflammatory effects were analyzed using Western blot, enzyme-linked immunosorbent assay (ELISA), and immunofluorescence, focusing on the role of hsa-let-7g-5p in regulating the nuclear factor κB (NF-κB)/NOD-like receptor protein 3 (NLRP3) pathway.

**Results:** The UCMSC-NVs acquired through serial extrusion displayed bilayer vesicle structures, with a mean diameter of around 100 nm. Moreover, these vesicles exhibited elevated expression levels of CD9, CD63, and CD81. Administration of UCMSC-NVs significantly alleviated lung damage, accompanied by a reduction in alveolar leakage and neutrophil infiltration. Furthermore, it significantly downregulated the expression of pro-inflammatory cytokines, such as interleukin (IL)-6, IL-1β, and tumor necrosis factor-α (TNF-α). UCMSC-NVs reduced macrophage infiltration in the lungs of ALI mice by inhibiting the NF-κB/NLRP3 signaling, leading to a shift in macrophage polarization, with decreased M1 and increased M2 polarization. Our findings demonstrated a novel therapeutic mechanism wherein hsa-let-7g-5p encapsulated within UCMSC-NVs alleviates inflammation by inhibiting NF-κB/NLRP3 expression, thereby mitigating ALI.

**Conclusion:** These results provide a foundation for the development of novel cell-free therapies with clinical potential for treating inflammatory lung diseases such as ALI and acute respiratory distress syndrome (ARDS).

## INTRODUCTION

Acute lung injury (ALI), typified by intense inflammation and pulmonary tissue damage, poses a considerable health challenge, associated with high rates of illness and death. The intricate pathophysiological process of ALI, frequently initiated by infections, sepsis, gastric content aspiration, or injury, encompasses a series of inflammatory reactions that result in the migration of neutrophils, macrophages, and additional immune cells into the affected areas^[1-5]^. These inflammatory cells emit reactive oxygen species (ROS), proteases, and cytokines, inflicting damage on the lung parenchyma. Consequently, this leads to heightened vascular permeability, the development of alveolar edema, and compromised gas exchange capabilities. Current therapeutic approaches to ALI are largely supportive, aiming to maintain adequate oxygenation, fluid balance, and organ function. However, the lack of specific therapies targeting the underlying inflammatory processes limits the effectiveness of treatment.

Over the past few years, mesenchymal stem cells (MSCs), especially those derived from the human umbilical cord, known as UCMSCs, have emerged as a beacon of hope in the realm of regenerative medicine. This is attributed to their remarkable ability to self-renew, differentiate into multiple lineages, and engage in paracrine signaling activities. Among the various secretory products of UCMSCs, exosomes have emerged as potent therapeutic agents^[6,7]^. These nanovesicles carry a range of bioactive molecules, including microRNAs (miRNAs), growth factors, and cytokines, which can modulate inflammatory responses, promote tissue repair, and inhibit apoptosis^[8,9]^. Stem cell-derived exosomes have shown promise in clinical applications due to their low immunogenicity, ease of preservation, and ethical advantages. However, they face challenges such as low yield, complex purification processes, and lack of unified quality standards. To overcome these, serial extrusion has emerged as a promising method for producing nanovesicles with high yield and similarity to exosomes. Our previous work found that UCMSC-NVs increased yield by over 20 times and exhibited equivalent effects to exosomes in a mouse model of diabetic chronic wounds^[10]^. Nanovesicles derived from MSCs through serial extrusion have been reported to suppress inflammatory cytokine secretion and infiltration of neutrophils and monocytes in the peritoneum of septic mouse models induced by outer membrane vesicles (OMVs), primarily by upregulating interleukin (IL)-10^[11]^. Recent studies have underscored the pivotal functions of miRNAs across a wide spectrum of physiological and pathological events. The miRNAs contained in nanovesicles derived from stem cells, which are membranous structures with diameters spanning from 40 to 200 nm, can be taken up by target cells. This uptake enables them to modulate responses in both local and distant organs and tissues^[12,13]^. In addition, miRNAs play essential roles in regulating lung development, maintaining pulmonary cell populations, and participating in lung injury and repair processes^[[14]]^. Research has indicated that exosomes derived from human umbilical cord mesenchymal stem cells (hucMSCs) are capable of inducing autophagy by targeting regulatory associated protein of MTOR complex 1 (RPTOR) through miR-377-3p. This process results in a reduction of inflammatory cytokine production and aids in the repair of ALI^[15]^. A recent investigation indicates that miRNAs contained in extracellular vesicles, which are released by human bronchial epithelial cells, can mitigate fibrosis. This mitigation is achieved through the suppression of Wingless/Integrated (WNT) signaling and the inhibition of transforming growth factor-β (TGF-β)-induced myofibroblast differentiation and the onset of cellular senescence^[16]^. Given these promising findings, EVs are considered potential therapeutic candidates for mitigating ALI, and their intrinsic regulatory mechanisms deserve further investigation. In this study, we hypothesize that the protective effects of UCMSC-NVs in treating ALI are potentially mediated through miRNA-directed anti-inflammatory mechanisms.

Several inflammatory signaling pathways are involved in the regulation of acute pulmonary inflammation and injury, with the nuclear factor κB (NF-κB) pathway being widely recognized as a pivotal contributor^[17]^. Prior research has shown that suppressing NF-κB expression results in decreased release of pulmonary inflammatory cytokines, attenuation of pulmonary inflammation, and an improvement in survival rates in mice with lipopolysaccharide (LPS)-induced ALI^[18,19]^. Additionally, stem cells derived from menstrual blood have demonstrated the ability to mitigate acute pulmonary inflammation and injury. This is achieved through the delivery of miR-671-5p via extracellular vesicles. The subsequent action of miR-671-5p is to target adaptor-associated protein kinase 1 (AAK1), thereby inhibiting the activation of the NF-κB signaling pathway, as reported in a previous study^[20]^. However, the potential of UCMSC-NVs to similarly regulate the NF-κB pathway in ALI remains unexplored. Our hypothesis posits that the therapeutic benefits of UCMSC-NVs against ALI are likely orchestrated through anti-inflammatory pathways governed by miRNAs. Through bioinformatics screening, a putative interaction site was detected between the 3′ untranslated region (3′ UTR) of the REL gene (REL oncogene, a NF-κB subunit gene), a key player in the NF-κB transcription factor family, and the miRNA hsa-let-7g-5p. This finding implies that hsa-let-7g-5p may serve as a novel regulator in the NF-κB signaling pathway, potentially alleviating the severity of ALI.

This research endeavor aimed to explore the therapeutic potential of UCMSC-NVs in combating ALI, with a particular emphasis on their ability to block the NF-κB/NOD-like receptor protein 3 (NLRP3) pathway. Our goal was to evaluate the protective effect of UCMSC-NVs on LPS-triggered ALI and to clarify the mechanisms at play in both animal models and cell cultures. The findings revealed that UCMSC-NVs confer their defensive effects in ALI by transporting hsa-let-7g-5p, thereby repressing the NF-κB pathway and, as a consequence, inhibiting the activation of the NLRP3 inflammasome. To sum up, the present work sheds new light on the molecular processes involved in UCMSC-NVs-mediated attenuation of pulmonary inflammation, enhancing the potential of UCMSC-NVs in the realms of regenerative medicine and therapeutic interventions for diseases. These findings have the potential to pave the way for the development of innovative treatment strategies for ALI.

## METHODS

### Animals

Six-week-old male BALB/c mice, procured from GemPharmatech Co. Ltd. in Shanghai, China, were housed in a controlled, sterile setting. This environment maintained a stable temperature of 23-25 °C, with a relative humidity between 50% and 60%. Mice were maintained under a stable light-dark regimen, which alternated between 12 h of illumination and 12 h of darkness. They had unrestricted access to both food and water. All surgical procedures and experimental protocols were approved by the Ethics Committee of Zhejiang Provincial People’s Hospital (Approval No. 20241128153583). Prior to the commencement of experiments, the mice underwent a one-week acclimatization period, during which they were anesthetized using isoflurane inhalation. Every step involving the use of animals was executed following a set of exacting principles that were solely focused on alleviating the anguish and hardship endured by the creatures involved.

### Cell culture

The MH-S macrophage cell line from murine origin (catalog number ATCC CRL-2019) was propagated in Roswell Park Memorial Institute 1640 medium (RPMI 1640) medium (supplied by Amizona Scientific LLC). The medium was supplemented with 10% fetal bovine serum (FBS, Amizona Scientific LLC), along with a cocktail of antibiotics including 100 U/mL penicillin and 100 μg/mL streptomycin, both sourced from Gibco, USA. Before exposure to 100 ng/mL LPS (Sigma, USA), the MH-S cells were incubated with UCMSC-derived nanovesicles (UCMSC-NVs) at a density of 1 × 10^8^ particles per milliliter for a duration of 30 min. Subsequently, the effects of UCMSC-NVs on murine alveolar macrophages under LPS stimulation were assessed. Cells of the MH-S line were plated into six-well dishes at a rate of 2 × 10^5^ cells per well. Following their reaching a confluence of 70%-80%, the cells were subjected to LPS treatment to elicit an inflammatory response. Following this, the cells received a treatment of UCMSC-NVs at a concentration of 1 × 10^8^ particles per milliliter, in conjunction with the Lipofectamine® 3000 reagent (Thermo Fisher Scientific, Carlsbad, CA) for the transfection process involving hsa-let-7g-5p mimics and inhibitors. The treatments were maintained for 24 or 48 h to assess the corresponding changes in messenger RNA (mRNA) and protein levels, respectively.

### Preparation and characterization of UCMSC-NVs

UCMSCs were procured from Weway (Hangzhou) Biotechnology Co., Ltd., located in Hangzhou, China, with their authenticity verified by the company. The cells were cultured in MesenCult™ MSCs Basal Medium, further enhanced with MesenCult™ MSCs Stimulatory Supplement (a product of STEMCELL Technologies, Canada). Cultivation took place in 10 cm diameter dishes until the cells attained a density of roughly 70%-80% confluence. Subsequently, the culture medium was exchanged for a serum-free version, and the cells were incubated for an extended period of 24-48 h^[21]^. Next, the cells were harvested and diluted to achieve a density of 2 × 10^6^ cells per milliliter. The cell suspension was subjected to a filtration process using a cascade of polycarbonate filters with progressively smaller apertures, starting at 10 µm and decreasing to 5 µm, 1 µm, 400 nm, and finally 200 nm, with each filter being used for either 11 or 21 cycles. Post-extrusion, the vesicles were spun horizontally at 4 °C for a total of 10 min to facilitate the separation of the supernatant. This separated supernatant was then subjected to two additional centrifugation steps at 4 °C, first at 2,000 *g* for a period of 20 min, and then at 10,000 *g* for 30 min. The supernatant from these centrifugation steps was lastly passed through a filter with a pore size of 0.22 µm. Subsequently, a high-speed centrifugal separation was conducted at a force of 100,000 *g* and a temperature of 4 °C for 70 min, after which the upper fraction was discarded. The isolated nanovesicles were rehydrated in 50 µL of chilled phosphate-buffered saline (PBS) and subsequently preserved at a temperature of -80 °C.

The ultrastructure and morphology of UCMSC-NVs were investigated and verified using transmission electron microscopy (TEM). The size distribution and zeta potential were measured by the NanoCoulter counter (Resun Technology, Co., LTD, Shenzhen) in a particle-by-particle manner, and the nanopore chip with the measuring range of 60-200 nm was chosen in the experiment. Additionally, nanoflow cytometry (VDOBIOTECH, Suzhou, China) was utilized to detect the expression profiles of specific surface markers on the vesicles, including CD9, CD63, and CD81 (Absin, Shanghai).

### Uptake of UCMSC-NVs

Initially, to eliminate potential bacterial contamination, the nanovesicles underwent filtration through a 0.22 μm membrane (Bioland, China). Subsequently, for tracking purposes, the UCMSC-NVs were labeled with the green fluorescent dye 3,3′-dioctadecyloxacarbocyanine perchlorate (DIO; Beyotime, Shanghai, China). A 10 μM solution of DIO was introduced to the nanovesicle suspension. The mixture was then placed in the dark at ambient temperature for a 25-minute incubation. Following this incubation, ultracentrifugation was performed at a speed of 100,000 *g* for 70 min at 4 °C. The upper layer was discarded, and the sediment was reconstituted in a PBS solution. To ensure the removal of any unbound dye, the suspension underwent two washing steps with PBS. The DIO-labeled UCMSC-NVs were then co-incubated with MH-S cells for 24 h to assess their uptake. After the incubation, the cells were immobilized and subjected to a triple washing process with PBS. Subsequently, they were labeled with Hoechst dye (Beyotime, Shanghai, China) to highlight the nuclei. The internalization of the nanovesicles was then examined and assessed under a TCS SP8 confocal microscope (Leica, Germany).

### Construction and sequencing of miRNA libraries

Utilizing the RNeasy Plus Micro Kit from QIAGEN in Germany, total RNA was isolated from UCMSC-NVs, strictly adhering to the protocol provided by the manufacturer. Following extraction, the quality and integrity of the small RNA were carefully assessed with the aid of an Agilent 2100 Bioanalyzer, which is manufactured by Agilent in Santa Clara, California, USA. Afterward, the process of preparing the small RNA library was initiated. For quantification purposes. The analysis relied on the Agilent 2100 DNA Bioanalyzer in conjunction with the Quant-iT PicoGreen double-stranded DNA (dsDNA) Assay Kit from Life Technologies. For the final step, the NEBNext Multiplex Small RNA Library Prep Set for Illumina, distributed by Shanghai Personal Biotechnology Co., Ltd. in the Chinese city of Shanghai, was applied to create miRNA libraries.

### LPS-induced ALI model *in vivo*

To simulate ALI, male BALB/c mice, aged seven weeks and certified pathogen-free, were selected. Mice were randomly allocated to the experimental groups using a computer-generated randomization scheme prior to LPS challenge and subsequent treatments. Animals and samples were coded with anonymized IDs to ensure blinded outcome assessment and blinded data analysis. They were subjected to intratracheal delivery of a non-fatal dose of Escherichia coli O111 LPS (5 mg/kg, sourced from Sigma-Aldrich in the USA) while under sodium pentobarbital-induced sedation. The LPS was diluted in a mere 50 μL of PBS. After waiting 4 h, the mice were treated with UCMSC-NVs via the tail vein at a density of 2 × 10^10^/mL. The subsequent monitoring for signs of life and changes in behavior was conducted at regular four-hour marks. After a 24-hour observation period, euthanasia was carried out via intraperitoneal injection of pentobarbital, followed by the collection of lung tissue samples. To avoid any potential interference from bronchoalveolar lavage fluid (BALF) collection on histological analysis, a separate experiment was conducted specifically for BALF collection. A total of 1 mL of PBS was instilled intratracheally, followed by six sequential lavages for BALF collection. The supernatant obtained after centrifugation was utilized for protein concentration determination using the bicinchoninic acid assay (BCA) assay. The cellular precipitate collected was processed for cell counting. Flow cytometric analysis was employed to assess and quantify the distinct cell populations in the BALF, including alveolar macrophages, which were identified with anti-CD11c and anti-Siglec F antibodies, interstitial macrophages marked by anti-F4/80 and anti-CD11b antibodies, and neutrophils tagged with anti-CD11b and anti-lymphocyte antigen 6 complex, locus G6D (Ly6G) antibodies.

### Biodistribution of UCMSC-NVs *in vivo*

To evaluate the biodistribution of UCMSC-NVs in a murine model, the nanovesicles were fluorescently labeled with the 1,1′-dioctadecyl-3,3,3′,3′-tetramethylindotricarbocyanine iodide (DIR) and subsequently administered via tail vein injection. Specifically, UCMSC-NVs at a concentration of 2 × 10^10^/mL were incubated with 5 μL of 10 μM DIR solution at room temperature, protected from light, for a duration of 30 min. A 7-week-old male BALB/c mouse model of ALI was established by intratracheal instillation of 5 mg/kg LPS. Following LPS instillation, DIR-labeled UCMSC-NVs were administered intravenously. Imaging of the mice was conducted at multiple time points (0, 2, 4, 8, 12, and 24 h post-injection) using an IVIS (*in vivo* imaging system) Lumina Series III imaging system (PerkinElmer, USA). Subsequently, the organs were excised for *ex vivo* IVIS imaging analysis to further assess biodistribution. A control group was established using healthy BALB/c mice that received unlabeled UCMSC-NVs to serve as a comparison.

### Histological analysis

The evaluation of lung injury intensity commenced with the immersion of lung tissue snippets in a 4% paraformaldehyde fixative, progressed to their insertion into a paraffin matrix, and was capped off with a detailed hematoxylin and eosin (H&E) staining analysis to reveal cellular details. Utilizing a Leica microscope (Wetzlar, Germany), a total of 10-15 random fields were selected from 5-μm thick sections at a magnification 200×. The analyzed specimens were evaluated for the magnitude of ALI, concentrating on the proliferation of inflammatory cells and the observed thickening of the alveolar septa. The severity of lung injury was scored on a standardized scale ranging from 0 to 4: 0 = no injury; 1 = up to 25% localized injury; 2 = up to 50%; 3 = up to 75%; 4 = diffuse injury. The lung injury score for each individual mouse was established by computing the mean values from the lung tissue sections. This assessment was performed separately by two seasoned pathologists under blind conditions using coded slides to maintain objectivity and dependability.

### Measurement of pulmonary vascular permeability

Evaluation of lung vascular permeability involved the injection of Evans blue dye (25 mg/kg, sourced from Sigma-Aldrich) into the tail vein. After allowing a two-hour period to pass, the surplus dye within the pulmonary circulation was cleared by perfusing heparinized saline into the right ventricle of the heart. Following this procedure, the lung tissue was then excised and prepared for further analysis. To extract the Evans blue dye from the lung tissue, the samples were immersed in 1 mL of formamide and subjected to an incubation period of 18 h at a temperature of 60 °C. Post-extraction, the lung tissue underwent lavage with 5 mL of PBS. The samples were then homogenized in 1 mL of PBS and washed twice to ensure thorough removal of unbound dye. A centrifugal force of 5,000 *g* was applied for half an hour to isolate the supernatant, which held the extracted dye. The Evans blue content in the supernatant was quantified using dual-wavelength spectrophotometry at 620 nm. In a concurrent procedure, the trachea and esophagus were meticulously separated to obtain whole, hydrated lung tissue, which was promptly weighed to ascertain its initial wet mass. Subsequently, this lung tissue was subjected to incubation at a temperature of 60 °C for a duration of 48 h, with the aim of evaporating all moisture content. Following this step, a reweighing was conducted to establish the tissue’s dry mass. The permeability of the pulmonary vasculature was then evaluated by computing the ratio of the lung tissue’s wet mass to its dry mass. This ratio provides a quantitative measure of the extent of fluid accumulation in the lungs, serving as an indirect indicator of vascular permeability.

### Enzyme-linked immunosorbent assay

For mice that developed lung injury due to LPS exposure, an evaluation of the pro-inflammatory cytokines TNF-α, IL-6, and IL-1β (Absin, Shanghai), and the counterbalancing anti-inflammatory cytokine IL-10 (Absin, Shanghai), was conducted via enzyme-linked immunosorbent assay (ELISA) technology. The protocol for this measurement was stringently followed as outlined by the ELISA kit’s producer. The optical density was assessed at a wavelength of 450 nm, employing an Epoch microplate reader manufactured by BioTek in the United States.

### Western blotting

After the process of tissue homogenization, the samples were incubated on ice for a duration of 30 min in a chilled solution of radio immunoprecipitation assay (RIPA) buffer, which was enhanced with a mixture of phosphatase inhibitors and phenylmethylsulfonyl fluoride (PMSF) to guard against protein degradation. Thereafter, the lysed tissue extracts were diluted in a 1:5 ratio with a 5× protein sample buffer from Abclonal, China. The diluted samples were then subjected to a heat treatment at 100 °C for a total of 5 min to achieve full protein denaturation. For protein separation, the extracts were electrophoresed on 4%-20% sodium dodecyl sulfate-polyacrylamide gel electrophoresis (SDS-PAGE) gels, applying a steady voltage of 120 V and a current of 220 mA for an extended time of 90 min. The proteins, having undergone electrophoretic separation, were then moved to PVDF membranes, imported from Millipore in Germany. After the protein migration, a 10-minute room temperature incubation with a blocking reagent was performed to ensure membrane saturation. Following this, the membranes were kept at 4 °C throughout the night, alongside a panel of primary antibodies, each prepared at a specific dilution rate: anti-NLRP3 (1:1,000, Abclonal, Inc.), anti-IL-1β (1:1,000, Cell Signaling Technology), anti-IL-18 (1:1,000, Abclonal, Inc.), anti-caspase-1 (1:1,000, Abclonal, Inc.), anti-IκBα (1:1,000, Abclonal, Inc.), anti-phosphorylated IκBα (p-IκBα) (1:1,000, Sigma), anti-p65 (p65: nuclear factor kappa-light-chain-enhancer of activated B cells subunit p65) (1:1,000, Abclonal, Inc.), and anti-β-actin (1:1,000, Abclonal, Inc.). After the overnight incubation, the membranes were then treated for 2 h at room temperature with horseradish peroxidase (HRP)-labeled secondary antibodies (HuaBio, China). The immunoreactive bands were visualized using an enhanced chemiluminescence (ECL) substrate and imaged with a qTouch Western Blot Imager (RWD Life Science Co., Ltd., Shenzhen, China). To maintain uniformity and comparability across various samples, the protein levels were normalized to β-actin expression.

### Quantitative real-time polymerase chain reaction

Lung tissue RNA from the mice was harvested using the TRIzol (a total RNA extraction reagent) reagent, adhering to the protocol’s guidelines. One microgram of the RNA yield was used to synthesize complementary DNA (cDNA), a process that involved the use of the PrimeScript RT Kit, procured from Takara in Dalian, China. To quantify the cDNA, the 7500 Real-Time PCR System by Applied Biosystems was employed, with the SYBR Green detection method, also a product of Takara, facilitating the analysis. For the purpose of normalization and cross-comparison of quantitative real time polymerase chain reaction (qRT-PCR) outcomes, the expression data were aligned to β-actin or glyceraldehyde-3-phosphate dehydrogenase (GAPDH) levels. The stem-loop RT protocol was engaged for the conversion of miRNA into cDNA, making use of the Mir-X™ miRNA First-Strand Synthesis Kit by Takara. The cDNA specimens thus prepared were maintained at a low temperature of -20 °C for potential analytical needs. The qRT-PCR experiments were conducted using the TB Green® Premix Ex Taq™ II (Tli RNaseH Plus) reagent kit from Takara. The sequences for the primers utilized in this research can be found in Supplementary Table 1. The 2^-ΔΔCt^ calculation method was used to establish the relative expression levels, and U6 small nuclear RNA was used as the benchmark for normalizing miRNA expression data.

### Immunofluorescence staining

After incubating lung tissue sections with primary antibodies specific for CD86 and CD206 (Absin, Shanghai), a secondary staining step was conducted using goat anti-rabbit Goat anti-rabbit IgG (H&L) antibody (IgG H&L) antibodies conjugated with fluorescein isothiocyanate (FITC) or tetramethylrhodamine thiocyanate (TRITC). Following the secondary antibody staining, the nuclei of the cells within the tissue were counterstained with 4′,6-diamidino-2′-phenylindole (DAPI; Absin, Shanghai) to provide a clear distinction between nuclear and cytoplasmic structures. For visualization and analysis, the stained lung tissue sections were examined using a confocal microscope (Leica, Germany).

### mRNA sequencing

Sequencing of the cDNA, DNA, and small RNA libraries was performed on the Illumina platform by 10K Genomics (Shanghai, China). Library preparation initiated with the extraction of total RNA, followed by the selective enrichment of mRNA with poly-A tails using Oligo(dT) magnetic beads. The enriched mRNA fragments were then used as templates for first-strand cDNA synthesis, facilitated by the Moloney murine leukemia virus (M-MuLV) reverse transcriptase. The RNA strand was subsequently cleaved using RNase H, with the subsequent generation of the second cDNA strand facilitated by DNA polymerase I. The resultant dsDNA was subjected to a series of modifications including end repair, A-tail addition, and adapter conjugation. For the purpose of selecting the appropriate fragment size, AMPure XP (a purification system based on solid-phase reversible immobilization) beads were applied to extract cDNA fragments within the 370 to 420 base pair range. These fragments were then amplified through PCR, purified, and finally assembled into the comprehensive library ready for sequencing.

The Qubit 2.0 Fluorometer was the instrument of choice for quantifying the library’s concentration before it was brought to a standardized 1.5 ng/μL. The Agilent 2100 Bioanalyzer played a role in profiling the insert sizes of the libraries. To ensure the library count was adequate, a qRT-PCR method was applied to pinpoint the concentration, necessitating it to be over 1.5 nM. Once the quality control measures were satisfied, the libraries were aggregated, taking into account their respective concentrations and the data needs. They were then sequenced using paired-end 150 bp reads on the Illumina platform. The sequencing process was based on the Sequencing by Synthesis (SBS), where fluorescent signals were captured and converted into sequence information.

### Statistical analysis

GraphPad Prism version 10 was utilized for the analysis of the data. Each experimental protocol was conducted three times, and the outcomes are reported as average values with the addition of standard deviation (SD). For evaluating the disparities between two separate groups, normally distributed data was analyzed with the aid of the Student’s *t*-test, while for data that did not meet the criteria for parametric analysis, the Mann-Whitney *U* test was the preferred statistical approach. For analyzing more than two groups, one-way analysis of variance (ANOVA) was the method of choice. In instances where data followed a normal distribution, the Brown-Forsythe and Welch corrections to ANOVA were applied, complemented by Tukey’s or Dunnett’s T3 tests for post-hoc comparisons. For data that was not normally distributed, the Kruskal-Wallis test with Dunn’s post-test was used for comparative analysis. A significance level of *P* < 0.05 was deemed statistically significant.

## RESULTS

### Preparation and characterization of UCMSC-NVs

**Figure 1:**
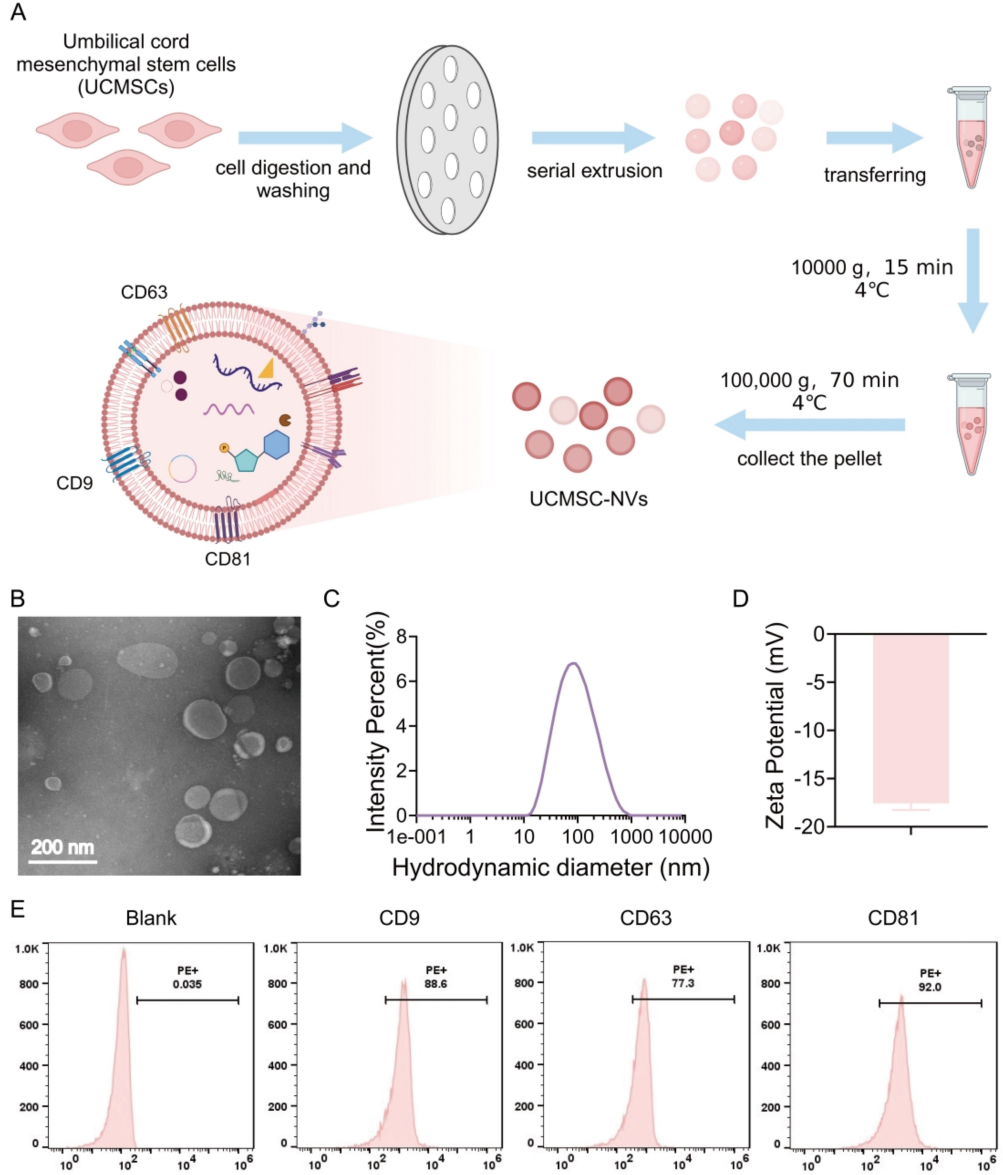
Preparation and Characterization of UCMSC-NVs. (A) Schematic illustration of the preparation of UCMSC-NVs through the serial extrusion of UCMSCs. Created in BioRender. Mou, X. (2026) https://app.biorender.com/illustrations/66b98605fa47a99541b0f630?slideId=72844bad-74f0-4878-9558-da4925fd93f8; (B) TEM characterization depicting the morphology and size of UCMSC-NVs; (C) DLS analysis illustrating the size distribution of UCMSC-NVs; (D) Zeta potential assessment of UCMSC-NVs; (E) Flow cytometry analysis of known. UCMSC-NVs: Nanovesicles originating from human umbilical cord mesenchymal stem cells; TEM: transmission electron microscopy; DLS: dynamic light scattering.

As shown in Figure 1A, the conditioned medium derived from UCMSCs was meticulously collected, followed by the isolation of nanovesicles through a process of serial extrusion. TEM was utilized to examine the morphological characteristics of the UCMSC-NVs. The TEM images revealed that these nanovesicles predominantly exhibit a spherical or elliptical shape, possessing a distinct bilayer membrane structure with diameters spanning from 90 to 150 nm [Figure 1B]. Additionally, dynamic light scattering (DLS) measurements substantiated that the nanovesicles had a size distribution with a mean diameter of approximately 100 nm [Figure 1C]. Our findings demonstrated that the zeta potential of UCMSC-NVs in PBS solution was -17.22 mV. This value signifies the stability of the nanovesicles in solution, as a negative zeta potential often indicates repulsive forces between particles, preventing aggregation [Figure 1D]. Additionally, flow cytometry analysis was conducted to assess the expression of specific surface markers. The results demonstrated a high positive expression of CD9 (92.53%), CD63 (93.61%), and CD81 (99.83%) on the surface of UCMSC-NVs, indicating the presence of these tetraspanins, which are commonly associated with exosomes and other extracellular vesicles [Figure 1E].

### Biodistribution of UCMSC-NVs

**Figure 2:**
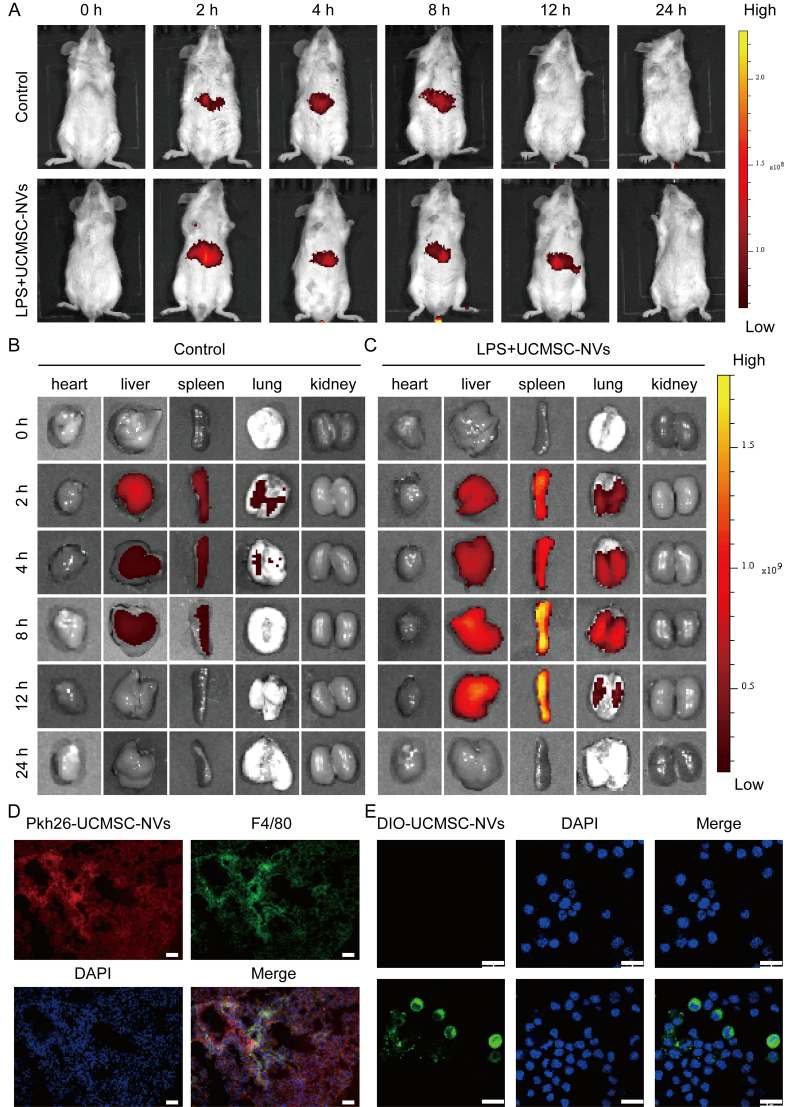
Biodistribution of UCMSC-NVs. (A) Fluorescence imaging of healthy and ALI mice at various time points following intravenous injection of DIR-UCMSC-NVs; (B) *Ex vivo* imaging of major organs from control mice; (C) *In vitro* visualization of key organs in ALI mice; (D) Uptake of PKH26-labeled UCMSC-NVs by pulmonary macrophages. Scale bar = 50 μm; (E) Confocal laser scanning microscopy showing intracellular localization of UCMSC-NVs following uptake. Scale bar = 20 μm. DIO-UCMSC-NVs: green, DAPI: blue. UCMSC-NVs: Nanovesicles originating from human umbilical cord mesenchymal stem cells; ALI: acute lung injury; DIR: 1,1′-dioctadecyl-3,3,3′,3′-tetramethylindotricarbocyanine iodide; PKH26: a red fluorescent membrane dye used for exosome labeling; DIO: 3,3′-dioctadecyloxacarbocyanine perchlorate; DAPI: 4′,6-diamidino-2′-phenylindole; LPS: lipopolysaccharide.

To assess the biodistribution of UCMSC-NVs *in vivo*, we utilized DIR dye [excitation wavelength (λEx)/emission wavelength (λEm) = 710/760 nm] for labeling the UCMSC-NVs, followed by intravenous administration via the tail vein of mice. The obtained results indicated a primary localization of DIR-labeled UCMSC-NVs within the abdominal region of the mice [Figure 2A]. Further analysis demonstrated the presence of detectable fluorescence signals from DIR-UCMSC-NVs in multiple organs, notably the liver, lungs, and spleen. In the group subjected to LPS treatment, DIR-UCMSC-NVs exhibited significant accumulation in lung tissue from 2 to 8 h post-injection, in contrast to the control group, indicating a targeted distribution pattern [Figure 2B and C]. To delve deeper into the uptake of UCMSC-NVs in pulmonary tissue, we assessed the distribution of PKH26 (a red fluorescent membrane dye used for exosome labeling)-labeled UCMSC-NVs four hours post-injection [Figure 2D]. Fluorescent imaging of cryosections from these lungs demonstrated extensive internalization of UCMSC-NVs by pulmonary macrophages at the four-hour time point [Figure 2D]. Additionally, in the *in vitro* study, UCMSC-NVs were labeled with DIO dye (λEx/λEm = 549/565 nm) and co-cultured with MH-S macrophages. The experimental results indicated that DIO-UCMSC-NVs were efficiently internalized by macrophages, predominantly accumulating within the cytoplasm compartment [Figure 2E]. These results collectively provide insight into the biodistribution and cellular internalization dynamics of UCMSC-NVs *in vivo*.

### UCMSC-NVs attenuate LPS-induced lung injury and reduce pulmonary vascular permeability

**Figure 3:**
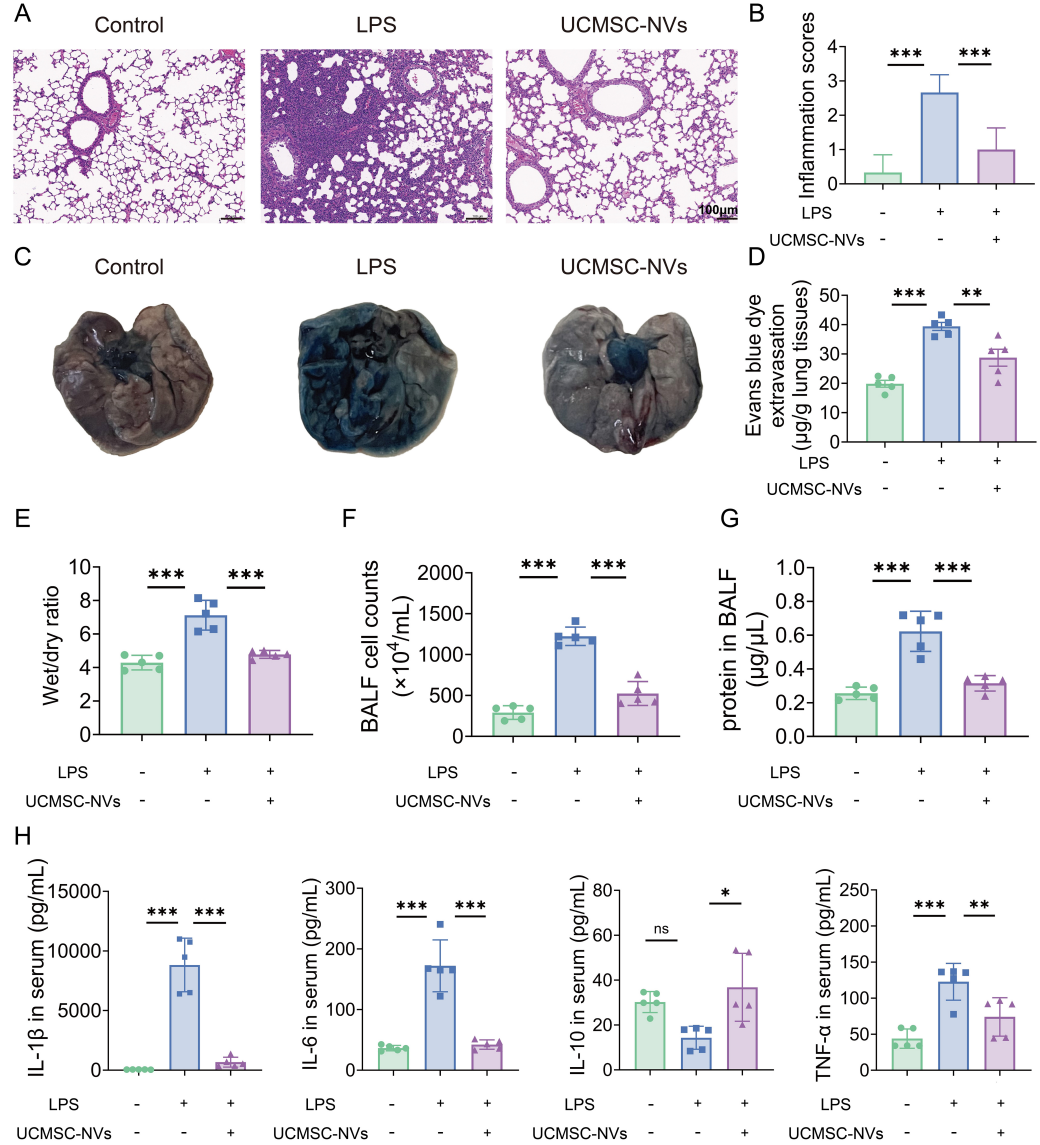
UCMSC-NVs attenuate LPS-induced lung injury and reduce pulmonary vascular permeability. (A) Impact of UCMSC-NVs on H&E-stained sections of lung tissue (scale bar = 100 µm); (B) Histopathological assessment of lung injury; *n* = 6 per group; (C) Accumulation of Evans blue dye within lung sections; (D) Spectrophotometric quantification of Evans blue extracted from lung tissue; *n* = 8 per group; (E) Ratio of W/D lung weights across various groups; *n* = 5 per group; (F) Enumeration of total cells in BALF; (G) Measurement of protein levels in BALF; *n* = 8 per group; (H) ELISA-determined concentrations of inflammatory cytokines (IL-6, IL-1β, TNF-α, and IL-10) in lung tissue extracts; *n* = 5 per group. Data represent means ± SD. Statistical analysis was performed by one-way ANOVA. ^*^P < 0.05, ^**^P < 0.01, ^***^P < 0.001, ns: not significant. UCMSC-NVs: Nanovesicles originating from human umbilical cord mesenchymal stem cells; LPS: lipopolysaccharide; H&E: hematoxylin and eosin; W/D: wet-to-dry; BALF: bronchoalveolar lavage fluid; ELISA: enzyme-linked immunosorbent assay; IL-6: interleukin-6; IL-1β: interleukin-1β; TNF-α: tumor necrosis factor-α; IL-10: interleukin-10; SD: standard deviation; ANOVA: analysis of variance.

Microscopic analysis of lung specimens in the LPS-treated group revealed extensive leukocyte infiltration, accompanied by diffuse interstitial and alveolar edema. Notably, there was significant thickening of alveolar septa, pulmonary hemorrhage, the formation of hyaline membrane within the alveolar spaces, and partial alveolar collapse. However, treatment with UCMSC-NVs markedly alleviated these pathological lung injuries, as depicted in Figure 3A. In contrast to the LPS group, the UCMSC-NVs-treated group exhibited a significant decrease in lung injury scores [Figure 3B]. As clearly demonstrated in Supplementary Figure 1, UCMSC-NVs and UCMSC-Exos exhibit similar therapeutic efficacy in the ALI mouse model, further supporting the translational potential of the serial extrusion method. To assess macromolecular permeability within the pulmonary vasculature, Evans blue dye staining was employed. LPS stimulation resulted in substantial leakage of Evans blue dye from the pulmonary vasculature into both the interstitial space and alveolar compartments [Figure 3C]. Conversely, UCMSC-NV treatment significantly mitigated this leakage [Figure 3D]. The wet-to-dry (W/D) weight ratio, a reliable indicator of pulmonary edema, demonstrated that the W/D ratio in the UCMSC-NVs-treated group approximated that observed in the control group [Figure 3E]. In contrast, mice exposed to LPS exhibited a significantly elevated W/D ratio. Subsequent investigations demonstrated that the administration of UCMSC-NVs led to a marked reduction in both the number of cells and protein content within BALF, as depicted in Figure 3F and G. The ELISA assays showed that, in comparison to the LPS-challenged group, the mice treated with UCMSC-NVs exhibited significantly reduced levels of the pro-inflammatory cytokines IL-6, IL-1β, and TNF-α. Concurrently, there was a marked increase in the levels of the anti-inflammatory cytokine IL-10 in these mice [Figure 3H].

### Comprehensive mRNA sequencing analysis of UCMSC-NVs-mediated gene expression and pathway modulations in ALI

**Figure 4:**
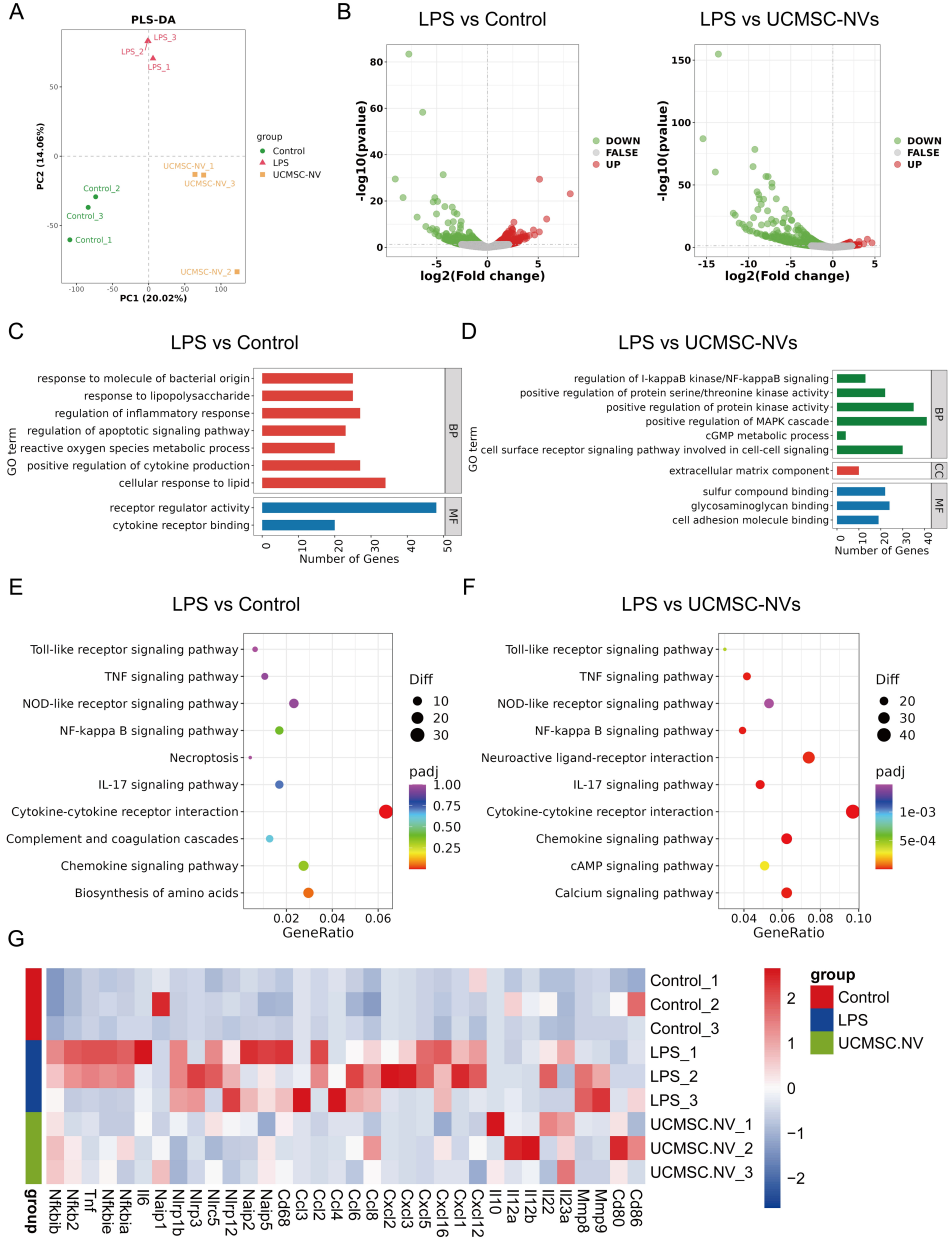
Comprehensive mRNA sequencing analysis of UCMSC-NV-mediated gene expression and pathway modulations in ALI. (A) PCoA visualizing the distribution among various mouse cohorts; (B) Volcano plots illustrating DEGs between the LPS and control groups, as well as between the LPS and UCMSC-NV treated groups; (C) GO enrichment analysis conducted for the LPS *vs.* control group comparison; (D) GO enrichment analysis for the LPS *vs.* UCMSC-NV group comparison; (E) KEGG pathway enrichment depicting pathways influenced by LPS exposure; (F) KEGG pathway enrichment identifying pathways modulated by UCMSC-NV treatment; (G) Heatmap depicting the expression fluctuations of key factors. mRNA: Messenger RNA; UCMSC-NV: nanovesicles originating from human umbilical cord mesenchymal stem cell; ALI: acute lung injury; PCoA: principal coordinate analysis; DEGs: differentially expressed genes; LPS: lipopolysaccharide; GO: Gene Ontology; KEGG: Kyoto Encyclopedia of Genes and Genomes; DA: dopamine; BP: biological process; MF: molecular function; CC: cellular component; MAPK: mitogen-activated protein kinase; cGMP: cyclic guanosine monophosphate; TNF: tumor necrosis factor; NOD: nucleotide-binding oligomerization domain; NF: nuclear factor; IL-17: interleukin-17.

In order to thoroughly investigate the effects of UCMSC-NVs on the RNA expression patterns in the lungs affected by ALI, we performed a genome-wide, high-capacity mRNA sequencing analysis. To analyze the variability between groups and the biological consistency within groups, we utilized principal coordinate analysis (PCoA). Ideally, a PCoA plot exhibits clear separation among groups and tight cohesion within each group. Our findings demonstrated robust reproducibility across groups, with statistically significant differences discernible between the normal, LPS-exposed, and UCMSC-NVs-treated groups [Figure 4A]. Transcriptome analysis identified 3,394 differentially expressed genes (DEGs) between the ALI model and normal groups, including 1,764 upregulated and 1,630 downregulated genes [Figure 4B]. Additionally, a comparison between the ALI model and UCMSC-NVs-treated groups revealed 1,803 DEGs, consisting of 642 upregulated and 1,161 downregulated genes [Figure 4B]. Gene Ontology (GO) functional enrichment analysis elucidated that LPS exposure primarily affected pathways associated with inflammation regulation, apoptotic signaling, and metabolism processes [Figure 4C]. Subsequent analyses were specifically tailored to focus on inflammation regulation. Figure 4D illustrates that following the treatment with UCMSC-NVs, a significant increase in the expression of genes linked to classic inflammatory signaling pathways, including the NF-κB and mitogen-activated protein kinase (MAPK) pathways, was observed. The subsequent examination via the Kyoto Encyclopedia of Genes and Genomes (KEGG) pathway enrichment validated the findings from the GO analysis, underscoring a significant enrichment in the NF-κB signaling pathways linked to inflammation [Figure 4E and F]. This indicates that UCMSC-NVs may alleviate LPS-induced pulmonary inflammation by modulating this pivotal pathway. As anticipated, the majority of inflammatory cytokines and chemokines, including NFKB inhibitor beta (Nfkbib), nuclear factor kappa B subunit 2 (Nfkb2), IL-6, IL-1β, TNF-α, matrix metallopeptidase 9 (Mmp9), C-C motif chemokine ligand 2 (Ccl2), C-C motif chemokine ligand 4 (Ccl4), and NLRP3, were significantly downregulated subsequent to UCMSC-NV treatment [Figure 4G]. Additionally, the protein-protein interaction (PPI) network presented in Supplementary Figure 2 shows 12 interconnected targets, including IL-6, TNF-α, Mmp9, IL-1β, Nfkbia, and NLRP3. These observations further substantiate that UCMSC-NVs may alleviate LPS-induced pulmonary inflammation by inhibiting the activation of the NF-κB pathway and NLRP3 inflammasome.

### UCMSC-NVs attenuate macrophage infiltration in ALI mouse lungs via inhibiting NF-κB/NLRP3 signaling pathway

**Figure 5:**
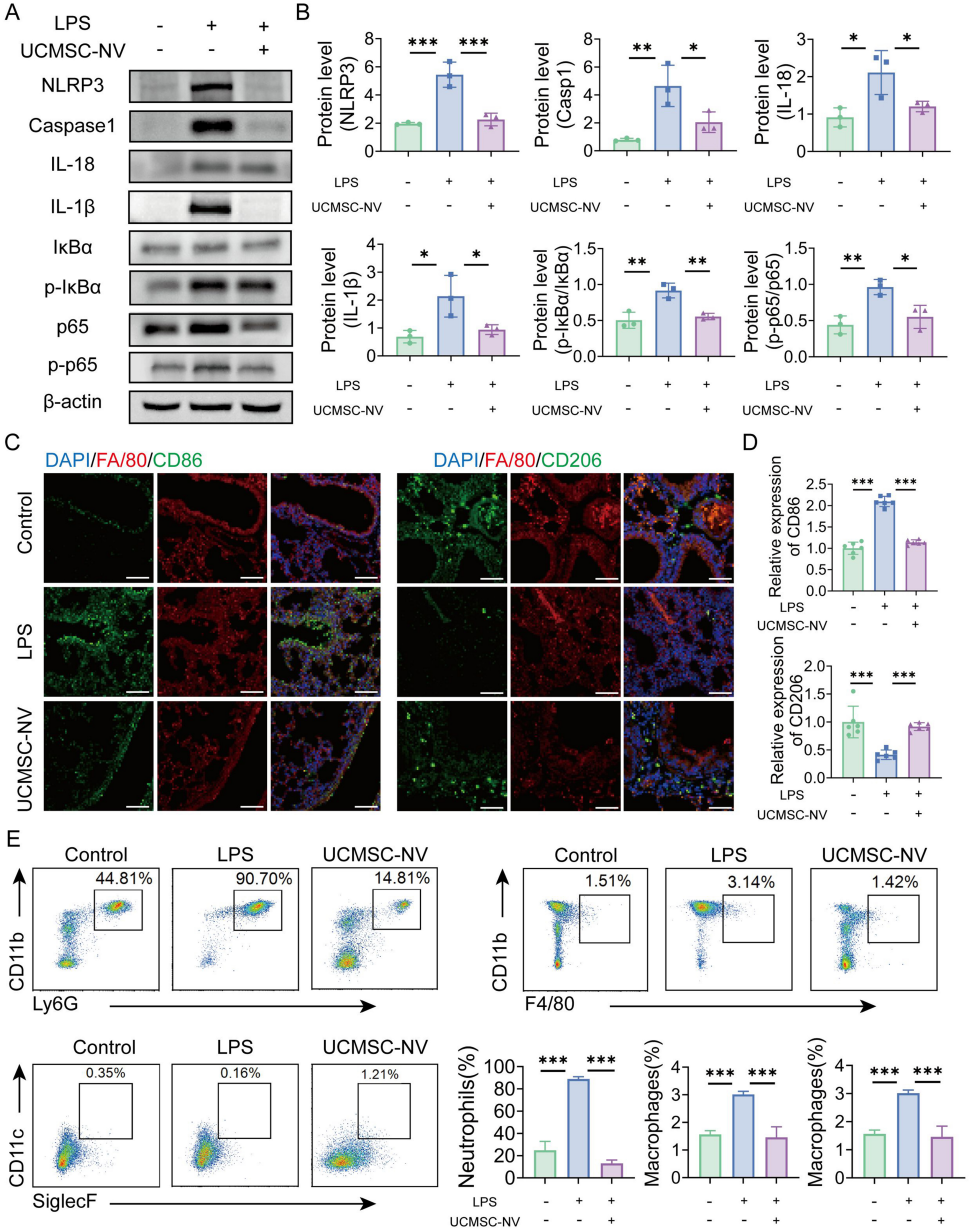
UCMSC-NVs attenuate macrophage infiltration in ALI mouse lungs via modulation of the NF-κB/NLRP3 signaling pathway. (A) Protein expression levels of IκBα, p-IκBα, total p65, p-p65, NLRP3, caspase-1, IL-18 and IL-1β were assessed by Western blotting; (B) Statistical analysis of histograms against the WB images in panel A. *n* = 3 per group; (C and D) Immunofluorescence analysis of M1 macrophage content in lung tissue (*n* = 6 per group) and M2 macrophage content in lung tissue. *n* = 6 per group. Scale bar = 100 μm; (E) Flow cytometry analysis of the proportions of neutrophils (CD11b+ Ly6G+) and macrophages (CD11b+ F4/80+) in BALF. *n* = 3 per group. Data represent means ± SD. Statistical analysis was performed by one-way ANOVA. ^*^P < 0.05, ^**^P < 0.01, ^***^P < 0.001. UCMSC-NVs: Nanovesicles originating from human umbilical cord mesenchymal stem cells; ALI: acute lung injury; NF-κB: nuclear factor κB; NLRP3: NOD-like receptor protein 3; p-IκBα: phosphorylated IκBα; p-p65: phosphorylated p65; IL-18: interleukin-18; IL-1β: interleukin-1β; WB: Western blot; BALF: bronchoalveolar lavage fluid; SD: standard deviation; ANOVA: analysis of variance; LPS: lipopolysaccharide; DAPI: 4′,6-diamidino-2′-phenylindole.

A thorough probe of the cytokine landscape within the lungs of LPS-exposed mice showed that treatment with UCMSC-NVs led to a dramatic reduction in IL-1β, IL-6, TNF-α, interferon-gamma (IFN-γ), matrix metallopeptidase 9 (MMP-9), and granulocyte-macrophage colony-stimulating factor (GM-CSF) levels [Supplementary Figure 3]. In stark contrast, mice affected by LPS-induced ALI showed a marked upsurge in the same cytokines, which highlighted a severe inflammatory episode within the pulmonary tissue [Supplementary Figure 3]. The LPS stimulus activated the NF-κB pathway, as seen by the enhanced ratio of phosphorylated p65 (p-p65) to total p65 and the augmented levels of p-IκBα compared to IκBα. Yet, the infusion of UCMSC-NVs markedly curtailed the p-p65/p65 ratio and suppressed the p-IκBα/IκBα levels [Figure 5A and B], signifying that UCMSC-NVs effectively repress the activation of NF-κB induced by LPS.

Additionally, mice with LPS-induced ALI showed a marked elevation in the percentage of F4/80+ CD86+ M1-type macrophages [Figure 5C and D]. In contrast, the UCMSC-NV-administered group saw a significant rise in the fraction of M2-like macrophages (F4/80+ CD206+), along with a marked decrease in the expression of the M1 macrophage marker CD86 [Figure 5C and D]. Consistently, immunofluorescence analysis further demonstrated that UCMSC-NV treatment decreased the M1 marker inducible nitric oxide synthase (iNOS) while restoring the M2 marker Arginase-1 in LPS-challenged lung tissue [Supplementary Figure 4]. UCMSC-NV treatment also significantly decreased the ratio of pro-inflammatory neutrophils and monocyte-derived macrophages [Figure 5E]. In summary, the findings suggest that UCMSC-NVs efficiently reduce macrophage infiltration in the pulmonary tissue of mice with ALI by repressing the NF-κB/NLRP3 pathway activation, decreasing M1 macrophage polarization, and fostering the shift towards M2 macrophage phenotype.

### UCMSC-NVs inhibit LPS-induced NF-κB/NLRP3 pathway activation *in vitro*

**Figure 6:**
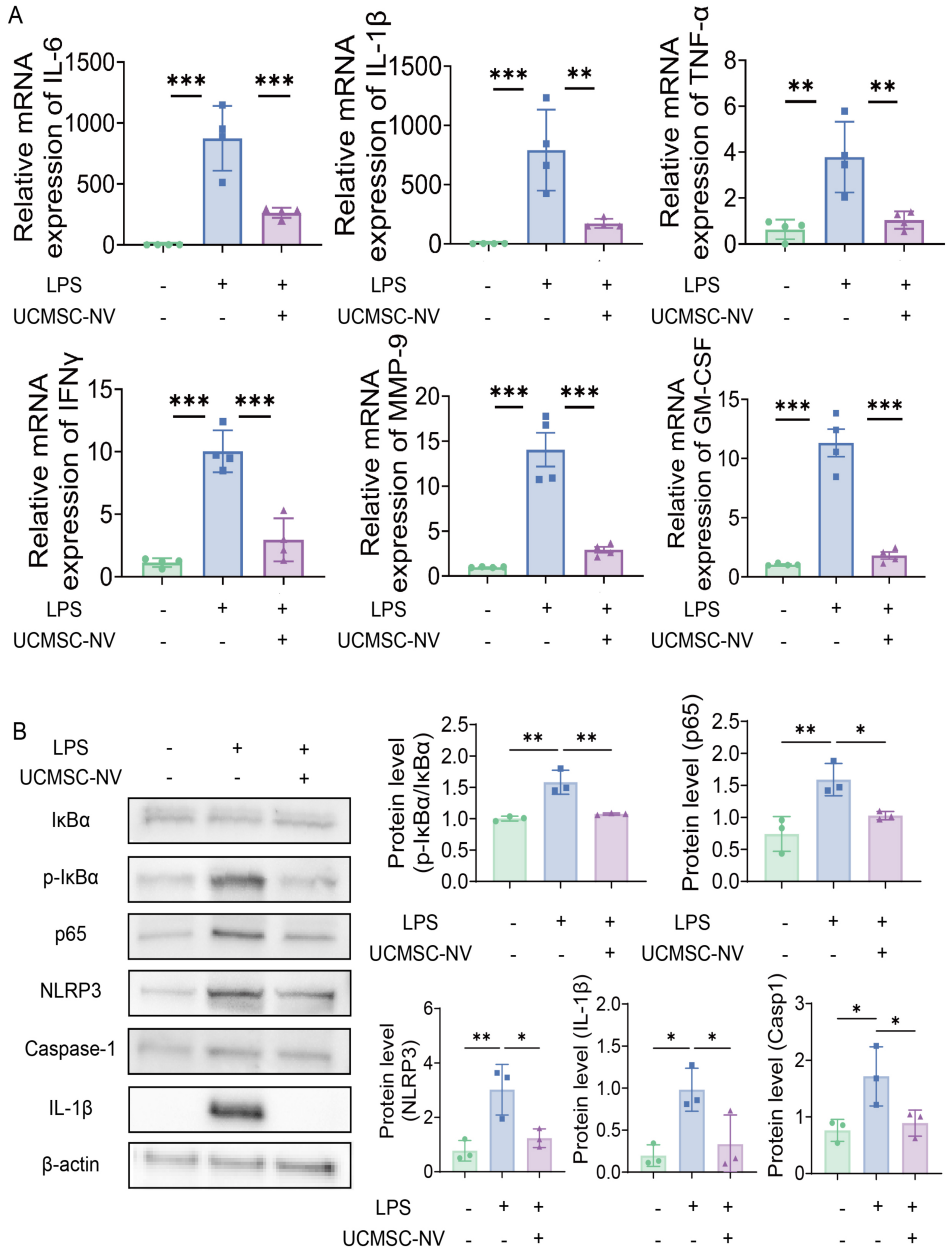
UCMSC-NVs inhibit LPS-induced activation of the NF-κB/NLRP3 pathway *in vitro*. (A) The transcriptional activity of pro-inflammatory cytokines in MH-S cells was monitored by quantitative PCR a day after LPS administration; (B) The protein profiles of phosphorylated and total p65, as well as phosphorylated and total IκBα in MH-S cells pretreated with UCMSC-NVs for half an hour before encountering LPS, were assayed using western blot methods. followed by LPS stimulation for 24 h. *n* = 3 per group; Western blot assessment of NLRP3, Caspase-1, and IL-1β protein expression levels. *n* = 3 per group. Data represent means ± SD. Statistical analysis was performed by one-way ANOVA. ^*^P < 0.05, ^**^P < 0.01, ^***^P < 0.001. UCMSC-NVs: Nanovesicles originating from human umbilical cord mesenchymal stem cells; LPS: lipopolysaccharide; NF-κB: nuclear factor κB; NLRP3: NOD-like receptor protein 3; PCR: polymerase chain reaction; IL-1β: interleukin-1β; SD: standard deviation; ANOVA: analysis of variance; mRNA: messenger RNA; IL-6: interleukin-6; TNF-α: tumor necrosis factor-α; IFNγ: interferon-gamma; MMP-9: matrix metallopeptidase 9; GM-CSF: granulocyte-macrophage colony-stimulating factor; p-IκBα: phosphorylated IκBα.

To explore the fundamental processes causing the activation of the NF-κB/NLRP3 pathway in cells challenged with LPS, we undertook a comprehensive analysis. Using reverse transcription quantitative polymerase chain reaction (RT-qPCR) techniques, we witnessed a marked elevation in the transcription of several inflammation-related cytokines, including IL-1β, IL-6, TNF-α, IFN-γ, MMP-9, and GM-CSF, subsequent to LPS activation. Interestingly, the administration of UCMSC-NVs markedly attenuated the mRNA levels of these cytokines [Figure 6A]. To further validate these findings, Western blot analysis was employed. This analysis confirmed that LPS stimulation led to a significant rise in the phosphorylation status of p65 (a subunit of c) and IκBα (inhibitor of NF-κB), evidenced by heightened ratios of phosphorylation to total proteins (p-p65/p65 and p-IκBα/IκBα) in macrophages. Importantly, UCMSC-NV treatment effectively downregulated the levels of these phosphorylated signaling proteins [Figure 6B]. In addition to these observations, we found that UCMSC-NVs significantly reduced the protein expression of NLRP3, a key component of the NLRP3 inflammasome. Furthermore, they inhibited the activation of Caspase-1 and the downstream maturation of IL-1β [Figure 6B]. Collectively, our findings suggest that UCMSC-NVs alleviate LPS-induced lung injury through the inhibition of the NF-κB/NLRP3 signaling pathway.

### hsa-let-7g-5p derived from UCMSC-NVs inhibits inflammation by targeting the NF-κB pathway

**Figure 7:**
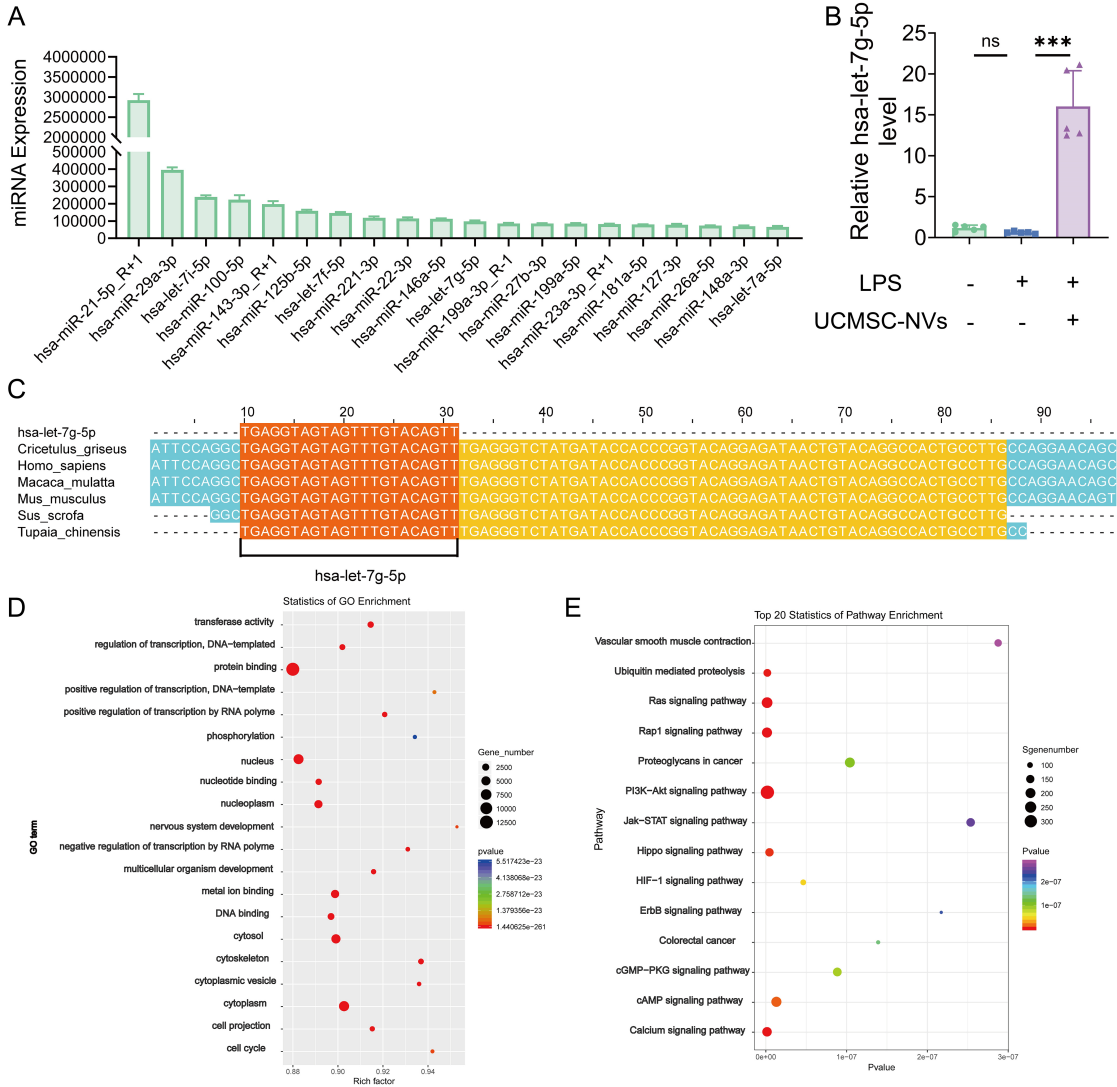
hsa-let-7g-5p as a key component of UCMSC-NVs regulates inflammation by targeting the NF-κB pathway. (A) Top 20 miRNAs identified in UCMSC-NVs; (B) RT-qPCR analysis of hsa-let-7g-5p expression in murine lung tissue. *n* = 5 per group. Data represent means ± SD. Statistical analysis was performed by one-way ANOVA. ^*^P < 0.05, ^**^P < 0.01, ^***^P < 0.001, ns: not significant; (C) Multiple species alignment illustrating the high sequence conservation of hsa-let-7g-5p across species; (D and E) GO and KEGG enrichment analyses of miRNAs. UCMSC-NVs: Nanovesicles originating from human umbilical cord mesenchymal stem cells; NF-κB: nuclear factor κB; miRNAs: microRNAs; RT-qPCR: reverse transcription quantitative polymerase chain reaction; SD: standard deviation; ANOVA: analysis of variance; GO: Gene Ontology; KEGG: Kyoto Encyclopedia of Genes and Genomes; LPS: lipopolysaccharide.

We provided a detailed elucidation of the effects and underlying mechanisms through which miRNAs derived from UCMSC-NVs influence ALI. A comprehensive profiling identified a spectrum of miRNAs, with a focused analysis on the top 20 most abundant miRNAs within UCMSC-NVs [Figure 7A]. Among them, hsa-let-7g-5p was detected in both UCMSC-derived exosomes and extrusion-derived NVs, with significantly higher levels in NVs (*P* < 0.0001), indicating preserved miRNA loading following extrusion [Supplementary Figure 5]. Subsequent quantitative PCR analysis demonstrated a significant upregulation of hsa-let-7g-5p expression in the lung tissue of ALI mice following UCMSC-NV treatment [Figure 7B]. It is worth highlighting that hsa-let-7g-5p shows strikingly conserved sequences among various species, which suggests its significant evolutionary value and likely crucial functions [Figure 7C]. This observation hints at a compensatory role for hsa-let-7g-5p in mitigating the progression of ALI. Moreover, to gain insights into the functional implications, we conducted GO, and KEGG enrichment analyses [Figure 7D and E]. These analyses revealed that hsa-let-7g-5p targets NF-κB, suggesting a mechanism by which hsa-let-7g-5p within UCMSC-NVs might exert anti-inflammatory effects by directly modulating NF-κB. In conclusion, our findings suggest that hsa-let-7g-5p serves as a pivotal regulatory molecule, mediating the protective effects of UCMSC-NVs in ALI by modulating inflammatory responses and intercellular communication pathways. This modulation contributes to alleviating LPS-induced lung injury. Our study supports the notion that UCMSC-NVs exert anti-inflammatory and tissue repair effects through the delivery of specific miRNA, thereby providing a molecular rationale for their potential application in the therapeutic management of ALI.

### hsa-let-7g-5p derived from UCMSC-NVs mediates anti-inflammatory effects through the NF-κB/NLRP3 pathway

**Figure 8:**
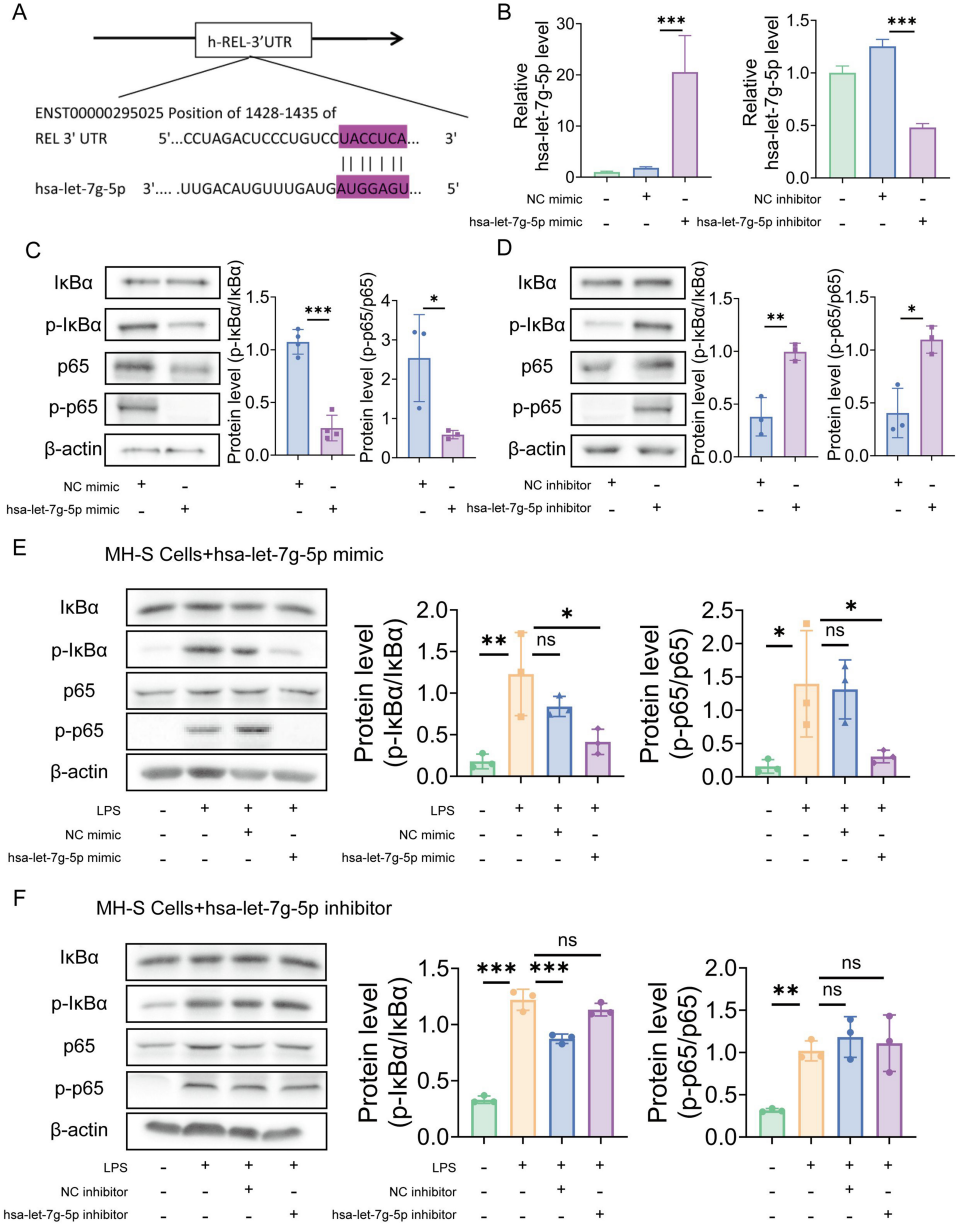
hsa-let-7g-5p in UCMSC-NVs mediates anti-inflammatory effects through the NF-κB/NLRP3 pathway. (A) Identification of two potential seed regions in the REL 3′ UTR; (B) Quantification of hsa-let-7g-5p expression levels via RT-qPCR. *n* = 3 per group; (C) Detection of NF-κB pathway protein levels via Western blotting following transfection with hsa-let-7g-5p mimic or NC mimic. *n* = 3 per group; (D) Detection of NF-κB pathway protein levels via Western blotting following transfection with hsa-let-7g-5p inhibitor or NC inhibitor. *n* = 3 per group; (E) Representative Western blot images of NF-κB pathway components in LPS-stimulated MH-S cells, 48 h post-transfection with hsa-let-7g-5p mimic or NC mimic. *n* = 3 per group; (F) Representative Western blot images of NF-κB pathway components in LPS-stimulated MH-S cells, 48 h post-treatment with hsa-let-7g-5p inhibitor or NC inhibitor. *n* = 3 per group. Data represent means ± SD. Statistical analysis was performed by one-way ANOVA. ^*^P < 0.05, ^**^P < 0.01, ^***^P < 0.001, ns: not significant. UCMSC-NVs: Nanovesicles originating from human umbilical cord mesenchymal stem cells; NF-κB: nuclear factor κB; NLRP3: NOD-like receptor protein 3; REL: REL oncogene, a NF-κB subunit gene; 3′ UTR: 3′ untranslated region; RT-qPCR: reverse transcription quantitative polymerase chain reaction; NC: negative controls; LPS: lipopolysaccharide; SD: standard deviation; ANOVA: analysis of variance; p-IκBα: phosphorylated IκBα; p-p65: phosphorylated p65.

To investigate the molecular foundations that govern the regulation of acute pulmonary inflammation and injury by hsa-let-7g-5p, we employed predictive algorithms to identify potential downstream targets. Notably, TargetScan and miRDB (a microRNA database), two miRNA prediction tools, identified REL as a likely target of hsa-let-7g-5p. REL, a critical transcriptional regulator within the NF-κB family, is essential in the adjustment of inflammatory reactions. This indicates that NF-κB might function as a principal downstream mediator of hsa-let-7g-5p in the control of lung inflammation and damage. Subsequent investigation exposed the existence of two highly effective seed sequences in the 3′ UTR of REL mRNA [Figure 8A], which serves as evidence for the participation of NF-κB in the hsa-let-7g-5p-induced reduction of pulmonary inflammatory and injury responses. To experimentally validate these findings, we performed transfections with Lipo3000 with hsa-let-7g-5p mimics or inhibitors, along with their respective negative controls (NC) mimics and inhibitors. Following this, the expression of hsa-let-7g-5p was measured through RT-qPCR. The data from the RT-qPCR analysis revealed no substantial variation in hsa-let-7g-5p levels between the NC mimic cohort and the control cohort. In contrast, the hsa-let-7g-5p mimic group exhibited a marked increase in hsa-let-7g-5p [Figure 8B].

Similarly, no significant difference in hsa-let-7g-5p levels was observed between the NC inhibitor and control groups, whereas a notable reduction was detected in the hsa-let-7g-5p inhibitor group. We sought to validate NF-κB as a direct target of hsa-let-7g-5p action by employing Western blot analysis to quantify the ratios of p-p65 to its total form and p-IκBα to total IκBα in macrophages after transfection with hsa-let-7g-5p mimics or inhibitors. The data revealed a statistically significant decrease in the p-p65/p65 and p-IκBα/IκBα ratios in the mimics-transfected cells, contrasted with an upward trend in the inhibitors-transfected cells [Figure 8C and D]. These findings underscore the role of hsa-let-7g-5p in suppressing NF-κB expression in macrophages through post-transcriptional regulation. We then probed into the influence of hsa-let-7g-5p on the NF-κB signaling pathway within macrophages challenged with inflammatory agents. The Western blot findings showed a significant elevation in the p-p65 to total p65 and p-IκBα to total IκBα ratios following LPS induction. However, transfection with hsa-let-7g-5p mimic effectively downregulated these proteins [Figure 8E]. *In vitro* assays further demonstrated that treatment with the NC inhibitor led to a marked increase in the levels of p-p65/p65 and p-IκBα/IκBα, which was not reversed by treatment with the hsa-let-7g-5p inhibitor [Figure 8F]. Collectively, these findings elucidate the critical role of the hsa-let-7g-5p/NF-κB axis in mediating the anti-inflammatory effects of UCMSC-NVs. Hsa-let-7g-5p acts as a crucial regulator in the control of pulmonary inflammation and injury by post-transcriptionally targeting and adjusting NF-κB expression.

### *In vivo* biosafety assessment of UCMSC-NVs

**Figure 9:**
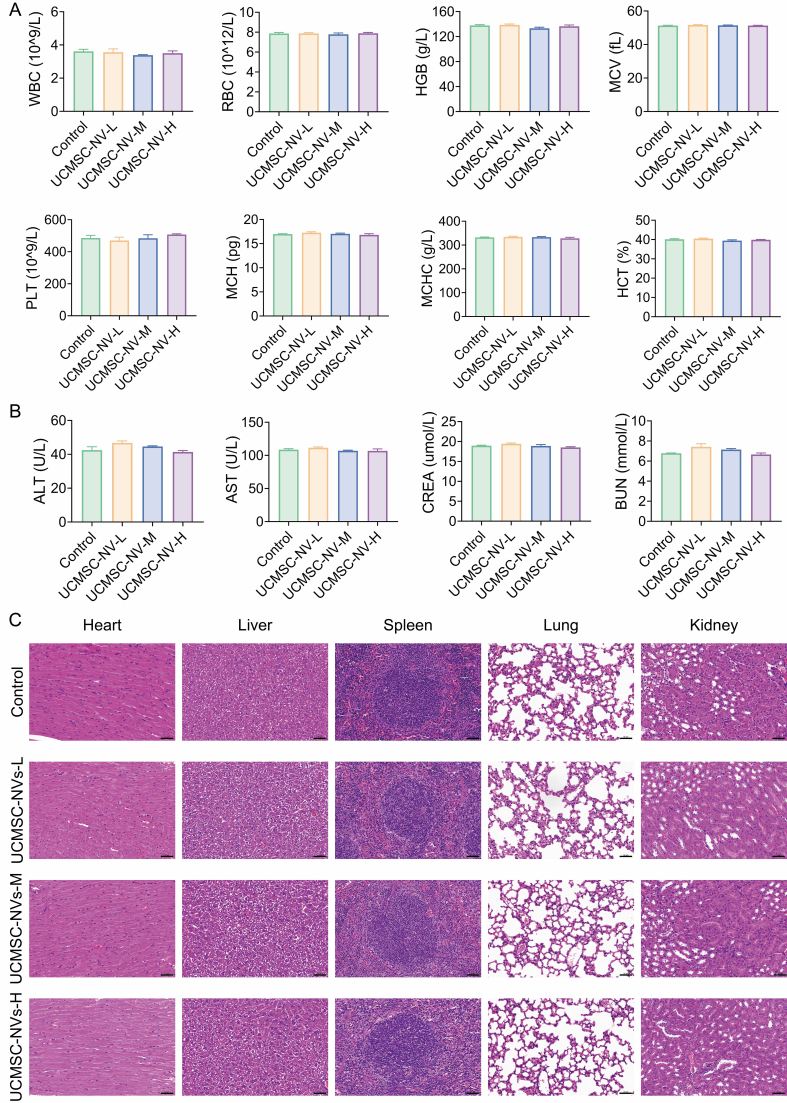
*In vivo* biosafety assessment of UCMSC-NVs. (A) Blood routine (*n* = 3 per group) of mice after different treatments; (B) Biochemical analysis (*n* = 3 per group) of mice after different treatments; (C) H&E staining of heart, liver, spleen, lung and kidney of mice after different treatments. UCMSC-NVs: Nanovesicles originating from human umbilical cord mesenchymal stem cells; H&E: hematoxylin and eosin; WBC: white blood cells; RBC: red blood cells; HGB: hemoglobin; MCV: mean cell volume; PLT: blood platelets; MCH: mean corpuscular hemoglobin; MCHC: mean corpuscular hemoglobin concentration; HCT: hematocrit. ALT: alanine transferase; AST: aspartate transferase; CREA: creatinine; BUN: blood urea nitrogen.

Therapeutic efficacy and biosafety represent fundamental prerequisites for the clinical translation of therapeutic agents. A comprehensive biosafety evaluation was first conducted using various doses of UCMSC-NVs. Male BALB/c mice (6 weeks old) received a single intravenous injection of 100 μL UCMSC-NVs at concentrations of 1 × 10^8^, 1 × 10^9^, and 1 × 10^10^ particles/mL, and were euthanized 24 h post-injection. No significant differences in hematological or biochemical parameters were observed between treatment and control groups, with all values remaining within normal reference ranges [Figure 9A and B]. Additionally, histological examination of the heart, liver, spleen, lungs, and kidneys revealed no apparent tissue damage or pathological changes in either group [Figure 9C]. These findings suggest that UCMSC-NVs possess a favorable biosafety profile, without evidence of hematotoxicity or organ toxicity.

## DISCUSSION

Initially, our study confirms that UCMSC-NVs confer significant therapeutic advantages in the context of ALI, efficiently repressing inflammatory responses, diminishing alveolar permeability, and curbing neutrophil infiltration. Moreover, we have delineated the modulatory function of hsa-let-7g-5p within UCMSC-NVs in the regulation of pulmonary inflammation. In addition, we have pinpointed NF-κB as a direct molecular target of hsa-let-7g-5p, highlighting its critical role in adjusting the NF-κB/NLRP3 signaling axis and decreasing macrophage accumulation in the lungs affected by ALI. Collectively, these results suggest that hsa-let-7g-5p contained in UCMSC-NVs mitigates inflammation associated with ALI by regulating the NF-κB/NLRP3 pathway and improving the macrophage environment. This investigation furnishes strong empirical evidence for the therapeutic promise of UCMSC-NVs in the treatment of ALI.

Our study demonstrates that UCMSC-NVs represent an effective vehicle for nanovesicle-based acellular therapies aimed at mitigating ALI. Existing research has demonstrated that MSCs are pivotal in slowing the advancement of lung damage^[22,23]^. As multipotent cells with immunomodulatory properties, MSCs exhibit substantial regenerative capacity^[24]^. Given their ethical accessibility, high availability, low immunogenicity, and potent regenerative capacity, UCMSCs have emerged as an ideal candidate for nanovesicle-based therapies^[25]^. Based on these attributes, we selected UCMSCs to explore their precision repair potential at injury sites. UCMSC-NVs, an emerging acellular therapeutic strategy, effectively circumvent immune rejection^[26]^. Compared to intact stem cells, they demonstrate reduced immunogenicity, pose no tumorigenic risk and offer enhanced clinical safety, and diminished ethical concerns. UCMSC-NVs, which usually vary in size from 40 to 150 nm, contain a rich mixture of proteins, nucleic acids, and lipids originating from the donor stem cells^[27]^. For this investigation, it was noted that UCMSC-NVs produced substantial immunomodulatory and lung-protective impacts within the LPS-challenged murine model of ALI. Results showed that DIR-labeled UCMSC-NVs were internalized by pulmonary macrophages, inhibiting M1 polarization while promoting M2 polarization, thus significantly reducing macrophage infiltration in ALI lungs. UCMSC-NVs also attenuated neutrophil accumulation in alveolar and interstitial spaces, decreased protein levels in BALF, and lowered pro-inflammatory mediator levels in lung tissue, thereby significantly ameliorating LPS-induced lung injury in mice. Overall, UCMSC-NVs demonstrated notable anti-inflammatory potential in both *in vivo* and *in vitro* experiments, highlighting their promise as a treatment strategy for pulmonary inflammatory diseases due to their low immunogenicity. However, the precise anti-inflammatory mechanisms of UCMSC-NVs warrant further investigation. Studies have shown that UCMSC-NVs play a crucial role in modulating cellular functions by regulating specific miRNAs in recipient cells^[28-33]^. Recent evidence indicates that cell-derived nanovesicles, serving as mediators of paracrine signaling, deliver anti-inflammatory miRNAs to target cells, thereby suppressing pathological protein production and influencing the progression of ALI^[34]^. A key challenge for synthetic miRNAs is their rapid degradation by plasma ribonucleases; however, cell-derived nanovesicles protect miRNAs from such degradation^[35]^. In this study, we employed high-throughput microarray analysis to establish the miRNA profile of UCMSC-NVs and identified hsa-let-7g-5p as highly expressed. Evidence suggests that the high conservation of hsa-let-7g-5p across species renders it a pivotal regulatory factor in UCMSC-NV-mediated modulation of acute lung inflammation and injury repair. Our *in vitro* experiments further confirmed that hsa-let-7g-5p in UCMSC-NVs can inhibit LPS-induced inflammation in lung macrophages. Thus, hsa-let-7g-5p in UCMSC-NVs represents an effective therapeutic strategy to mitigate inflammation and injury induced by LPS or other pathogens.

While the aforementioned findings suggest that hsa-let-7g-5p has a crucial impact on regulating pulmonary inflammation and injury, the precise molecular mechanisms by which it mitigates lung inflammation and damage remain to be fully elucidated. Analysis via bioinformatics suggests that NF-κB is targeted by hsa-let-7g-5p, leading to the suppression of ALI. Ubiquitously present in animal cells, NF-κB serves as a pivotal mediator of pro-inflammatory signaling and is primarily composed of subunits including v-rel avian reticuloendotheliosis viral oncogene homolog A (RelA), also known as the p65 subunit of NF-κB, NF-κB2 (p52), NF-κB1 (p50), RelB (RELB proto-oncogene, a subunit of NF-κB), and c-Rel (a member of the NF-κB family)^[29,30]^. Under normal physiological conditions, IκB binds to NF-κB, inhibiting its activity. Following activation, the disassociation of IκB initiates the phosphorylation of NF-κB and its subsequent translocation to the nucleus, where it promotes the synthesis of pro-inflammatory cytokines^[31]^. The research demonstrates that the application of UCMSC-NV-hsa-let-7g-5p mimics leads to a notable reduction in the levels of IκBα, p-IκBα, and p65 within macrophages. In contrast, the use of hsa-let-7g-5p inhibitors results in an elevation of these protein expressions. Such observations suggest a potential anti-inflammatory role for hsa-let-7g-5p, which may be achieved through direct interaction with the NF-κB pathway. Additionally, it was observed that LPS stimulation markedly amplified the expression of NF-κB p65 and p-IκB in the lung tissues of ALI mice. This implies that LPS induces IκB phosphorylation, thereby activating the NF-κB signaling cascade and subsequently increasing the production of inflammatory cytokines, which in turn intensifies the inflammatory response. UCMSC-NV treatment markedly reduced IκB phosphorylation, thereby inhibiting NF-κB signaling and alleviating inflammation. Within the innate immune system, the NLRP3 inflammasome, which consists of NLRP3, Caspase-1, and ASC, forms an essential multimeric complex^[32]^. It has been previously established that this inflammasome plays a significant role in the development of LPS-induced ALI, as it governs the maturation and secretion of inflammatory cytokines^[33]^. Moreover, the NLRP3 inflammasome is recognized as a key player in inflammatory responses mediated by NF-κB^[35,36]^. Our investigation, encompassing both *in vitro* and *in vivo* experiments, has ascertained that LPS stimulation triggers the expression of NF-κB p-p65 and NLRP3, thereby activating the NF-κB/NLRP3 inflammasome pathway. Of particular importance, treatment with UCMSC-NVs has been shown to effectively inhibit the LPS-induced activation of this pathway. Consequently, we deduce that UCMSC-NVs alleviate lung injury in ALI mice by modulating the NF-κB/NLRP3 pathway via hsa-let-7g-5p.

Several limitations of this study should be acknowledged. First, our *in vivo* experiments were primarily conducted in an intratracheal LPS-induced ALI model with a relatively short observation window (24 h). Although this model recapitulates key inflammatory features of ALI, it may not fully represent the pathophysiological heterogeneity of clinical ARDS caused by different etiologies (e.g., bacterial pneumonia, viral infection, or sepsis) and varying disease phases. Second, only male BALB/c mice of a single age range were used; therefore, potential influences of sex, age, and host background on the therapeutic response to UCMSC-NVs were not systematically evaluated. In addition, sample sizes were determined based on prior experience and feasibility rather than a formal a priori power calculation, which may limit the statistical power for certain endpoints. Third, we tested one main dosing condition and administration route *in vivo*. More comprehensive optimization of dose, dosing frequency, and therapeutic time window remains necessary. Moreover, while fluorescence imaging suggested lung localization and macrophage uptake of UCMSC-NVs, quantitative pharmacokinetics, rigorous biodistribution quantification, and long-term safety assessments (including potential immunogenicity) were not performed and warrant future investigation. Finally, while our data support a central role for the hsa-let-7g-5p/REL/NF-κB/NLRP3 axis [Figures 7 and 8], a definitive *in vivo* loss-of-function experiment using hsa-let-7g-5p–depleted NVs was not conducted. Therefore, although the mechanistic evidence strongly suggests sufficiency, contributions from other NV cargos cannot be entirely excluded. Future studies will generate hsa-let-7g-5p–reduced NVs with rigorous characterization to formally assess causal specificity *in vivo*.

To sum up, the present research has effectively established a technique for the fabrication of UCMSC-NVs and has carried out an extensive assessment of their capacity to mitigate inflammation within a framework simulating LPS-induced ALI. The obtained findings elucidated that UCMSC-NVs exhibit potent suppressive effects on the secretion of pro-inflammatory cytokines, mitigate pulmonary inflammation, and ameliorate tissue damage. This efficacy is mediated by the regulation of hsa-let-7g-5p, which specifically targets the NF-κB transcription factor subunit REL and modulates the NF-κB/NLRP3 signaling pathway. The findings presented herein provide fresh molecular perspectives and highlight the potential of UCMSC-NVs as an effective therapeutic strategy for addressing ALI.

## DECLARATIONS

### Availability of data and materials

The raw data supporting the findings of this study are available within this Article and its Supplementary Materials. Further data are available from the corresponding authors upon request.

### Copyright

© The Author(s) 2026.
