# Supplementary material for: Human umbilical cord mesenchymal stem cell-derived nanovesicles ameliorate acute lung injury by hsa-let-7g-5p inhibition of NF-κB/NLRP3 pathway
Source: Extracell Vesicles Circ Nucl Acids. 2026 Apr 2;7(2):465–89. doi: 10.20517/evcna.2025.170 (PMC13174211; doi:10.20517/evcna.2025.170)
Supplement: Supplementary file 1 [file evcna-7-2-465-SupplementaryMaterials.pdf]

Supplementary Materials

Human umbilical cord mesenchymal stem cell-derived nanovesicles ameliorate acute lung injury by hsa-let-7g-5p inhibition of NF-κB/NLRP3 pathway

Yilin Huang[1,2,3,#], Yan Zeng[1,#], Ailin Wu[1,#], Yang Chen[1,2], Yuanhao Zhou[1], Youni Zhang[4], Hai Zou[5], Weijiao Fan[1], Xiaoyi Chen[1], Jinyang Chen[6], Jie Wang[7], Xianghong Yang[1], Xiaoru Chang[1,2], Xiaozhou Mou[1,2], Yuexing Tu[8]

[1]Center for Rehabilitation Medicine, Rehabilitation & Sports Medicine Research Institute of Zhejiang Province, Department of Rehabilitation Medicine, Translational Medicine Center, Zhejiang Provincial People's Hospital, Hangzhou Medical College, Hangzhou 310014, Zhejiang, China.
[2]College of Pharmacy, Hangzhou Medical College, Hangzhou 310059, Zhejiang, China.
[3]Department of Pharmacy, Hubei Hospital of Integrated Chinese & Western Medicine, Wuhan 430015, Hubei, China.
[4]Clinical Laboratory Department, Tiantai People's Hospital of Zhejiang Province (Tiantai Branch of Zhejiang Provincial People's Hospital), Hangzhou Medical College, Taizhou 317200, Zhejiang, China.
[5]Department of Emergency and Critical Care Medicine, Shanghai Pudong New Area People's Hospital, Shanghai 200120, China.
[6]Weway (Hangzhou) Biotechnology Co., Ltd., Hangzhou 310052, Zhejiang, China.
[7]EVital Bio (Hangzhou) Co., Ltd., Hangzhou 310056, Zhejiang, China.
[8]Department of Critical Care Medicine, Tongde Hospital of Zhejiang Province, Hangzhou 310012, Zhejiang, China.
[#]Authors contributed equally.

Correspondence to: Prof. Yuexing Tu, Department of Critical Care Medicine, Tongde Hospital of Zhejiang Province, Hangzhou 310012, Zhejiang, China. E-mail: 2024t060@zcmu.edu.cn; Dr. Xiaozhou Mou, Dr. Xiaoru Chang, Emergency and Critical Care Center, Intensive Care Unit, Clinical Research Institute, Zhejiang Provincial People's Hospital, Affiliated People's Hospital, Hangzhou Medical College, Hangzhou 310014, Zhejiang, China. E-mail: mouxz@zju.edu.cn;

changxiaoru@hmc.edu.cn

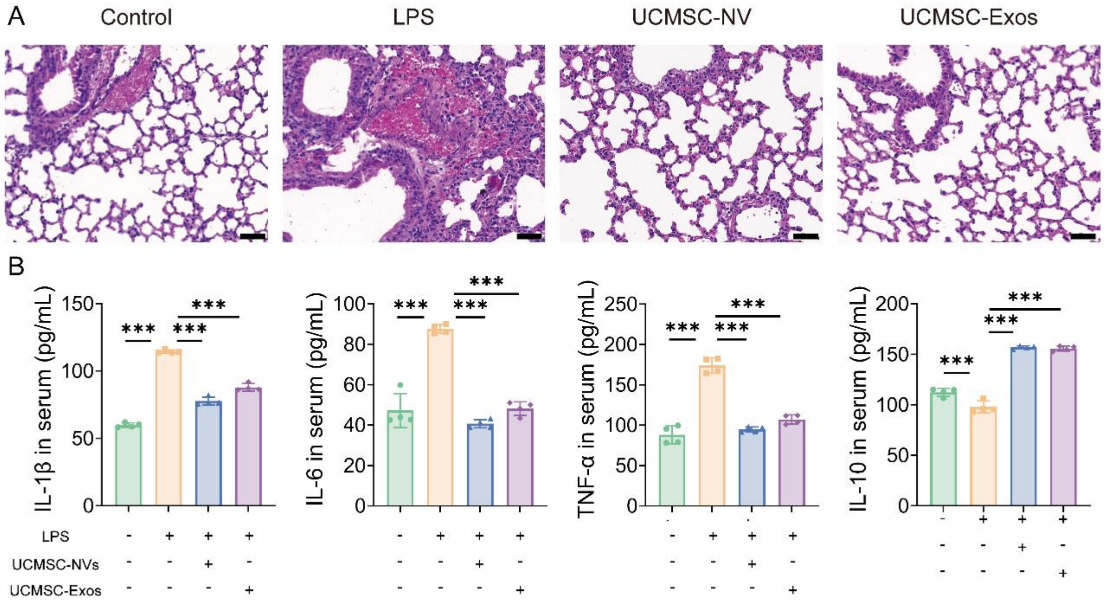

Supplementary Figure 1. UCMSC-NVs and UCMSC-Exos attenuate LPS-induced lung injury. (A) Impact of UCMSC-NVs and UCMSC-Exos on hematoxylin and eosin (H&E)-stained sections of lung tissue (scale bar = 100 μm); (B) ELISA-determined concentrations of inflammatory cytokines (IL-6, IL-1β, TNF-α, and IL-10) in lung tissue extracts; n = 4 per group. Data represent means ± SD. Statistical analysis was performed by one-way ANOVA. $*p < 0.05$, $**p < 0.01$, $***p < 0.001$, SD: standard deviation.

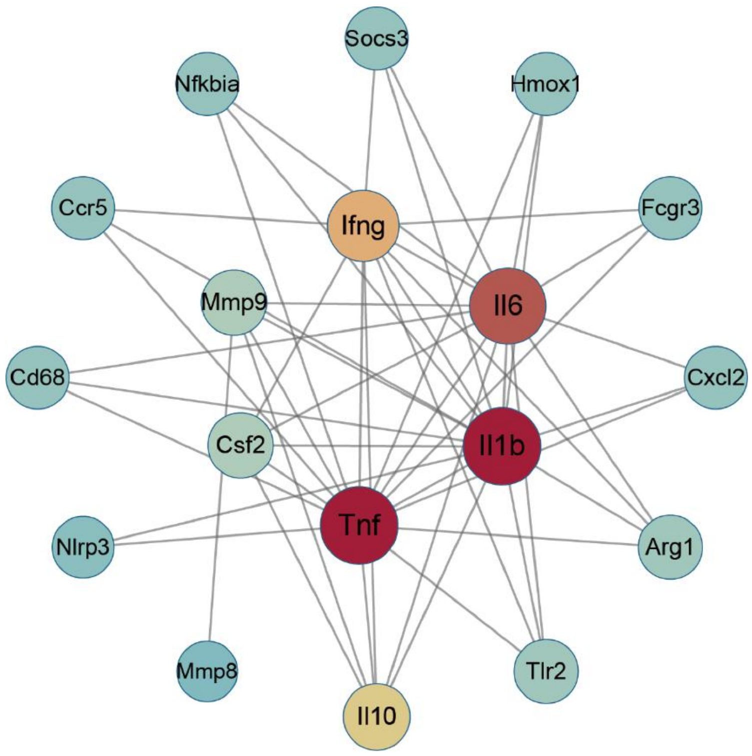

Supplementary Figure 2. UCMSC-NVs induce activation of the NF-κB/NLRP3 pathway.

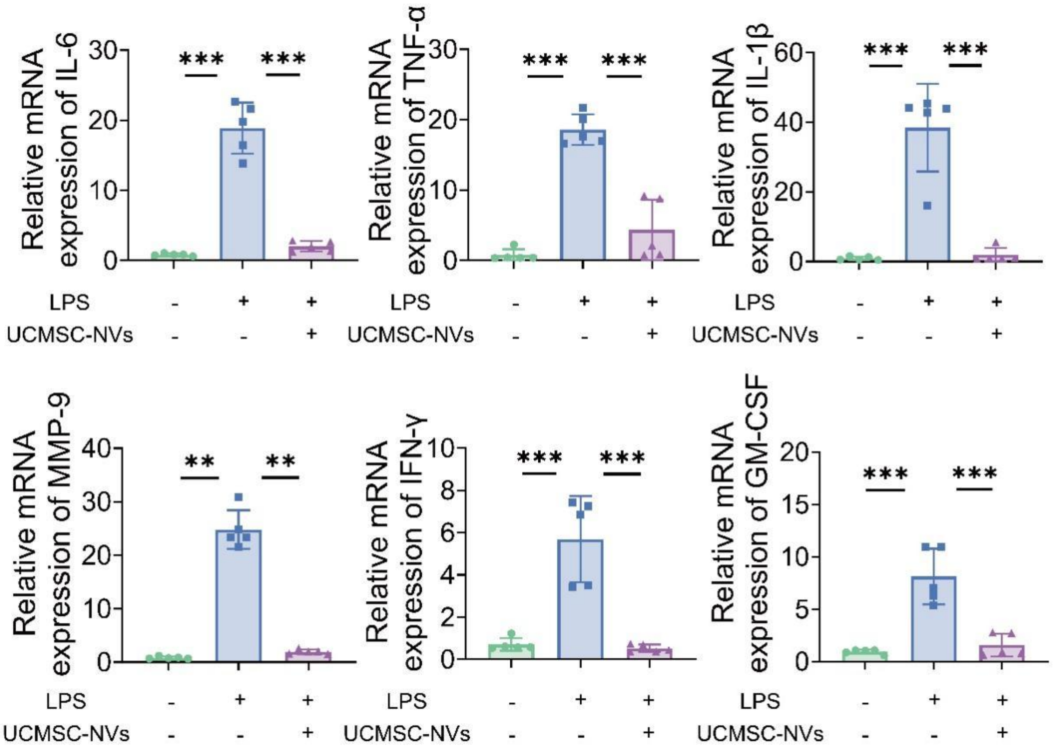

Supplementary Figure 3. RT-qPCR analysis shows the expression of inflammatory factors in the lung tissues of ALI mice treated with UCMSC-NVs. Data represent means ± SD. Statistical analysis was performed by one-way ANOVA. **p < 0.01, ***p < 0.001.

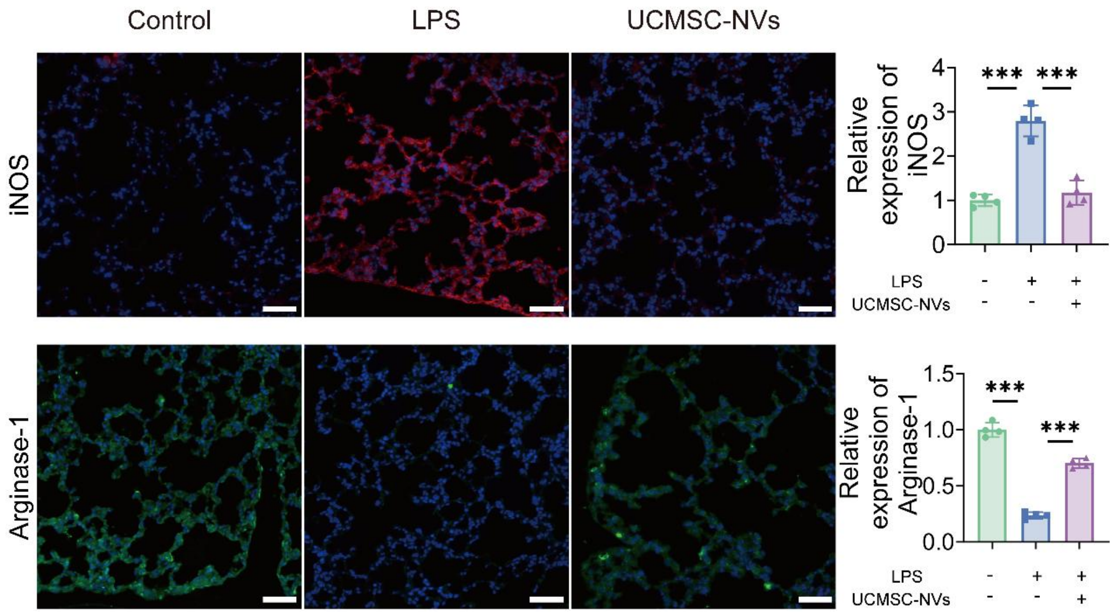

Supplementary Figure 4. Immunofluorescence analysis of M1 macrophage content in lung tissue (n = 4 per group) and M2 macrophage content in liver tissue. n = 4 per group. Scale bar = 100 μm. Data represent means ± SD. Statistical analysis was performed by one-way ANOVA. ***p < 0.001.

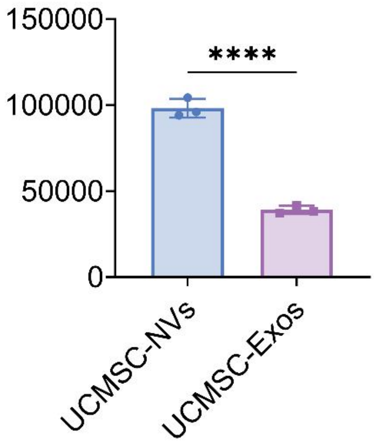

Supplementary Figure 5. hsa-let-7g-5p expression in UCMSC-NVs and UCMSC-Exos. n = 3 per group. Data represent means ± SD. Statistical analysis was performed by one-way ANOVA. ****p < 0.001.

Supplementary Table 1. List of real-time qPCR primer sets (mouse)

Gene	Forward primer (5′-3′)	Reverse primer(5′-3′)
IL-1β	TGCCACCTTTTGACAGTGATG	TGATGTGCTGCTGCGAGATT
IL-6	ACAAAGCCAGAGTCCTTCAGAG	TGTGACTCCAGCTTATCTCTTGG
TNF-α	TATGGCCCAGACCCTCACA	GGAGTAGACAAGGTACAACCCATC
β-actin	CACTGTCGAGTCGCGTCC	TCATCCATGGCGAACTGGTG
IFN-γ	CGGCACAGTCATTGAAAGCC	TGTCACCATCCTTTTGCCAGT
MMP-9	TGGTCTTCCCCAAAGACCTG	TAGCGGTACAAGTATGCCTCTG
GM-CSF	GCTAAGGTCCTGAGGAGGATG	CTACCTCTTCATTCAACGTGACA

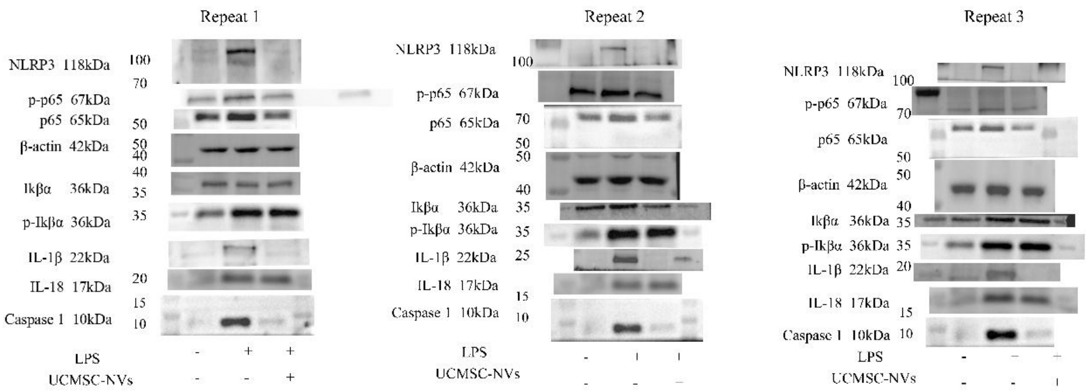

Supplementary Figure 6. Example of original western blot for three repeats (for Figure 5).

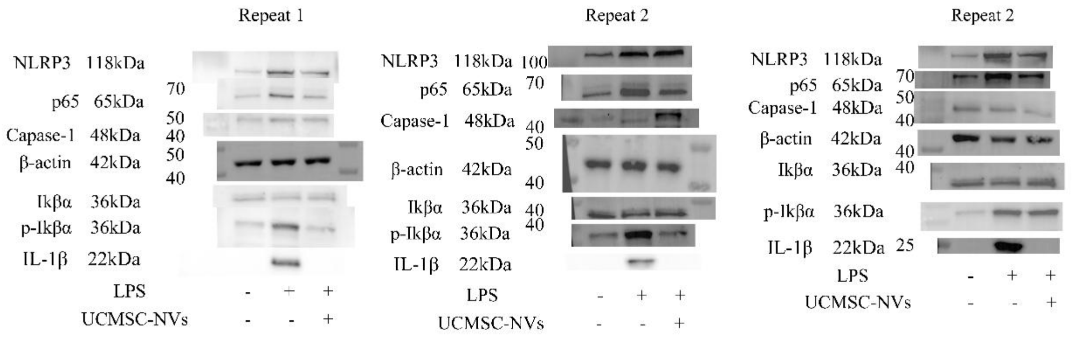

Supplementary Figure 7. Example of original western blot for three repeats (for Figure 6).

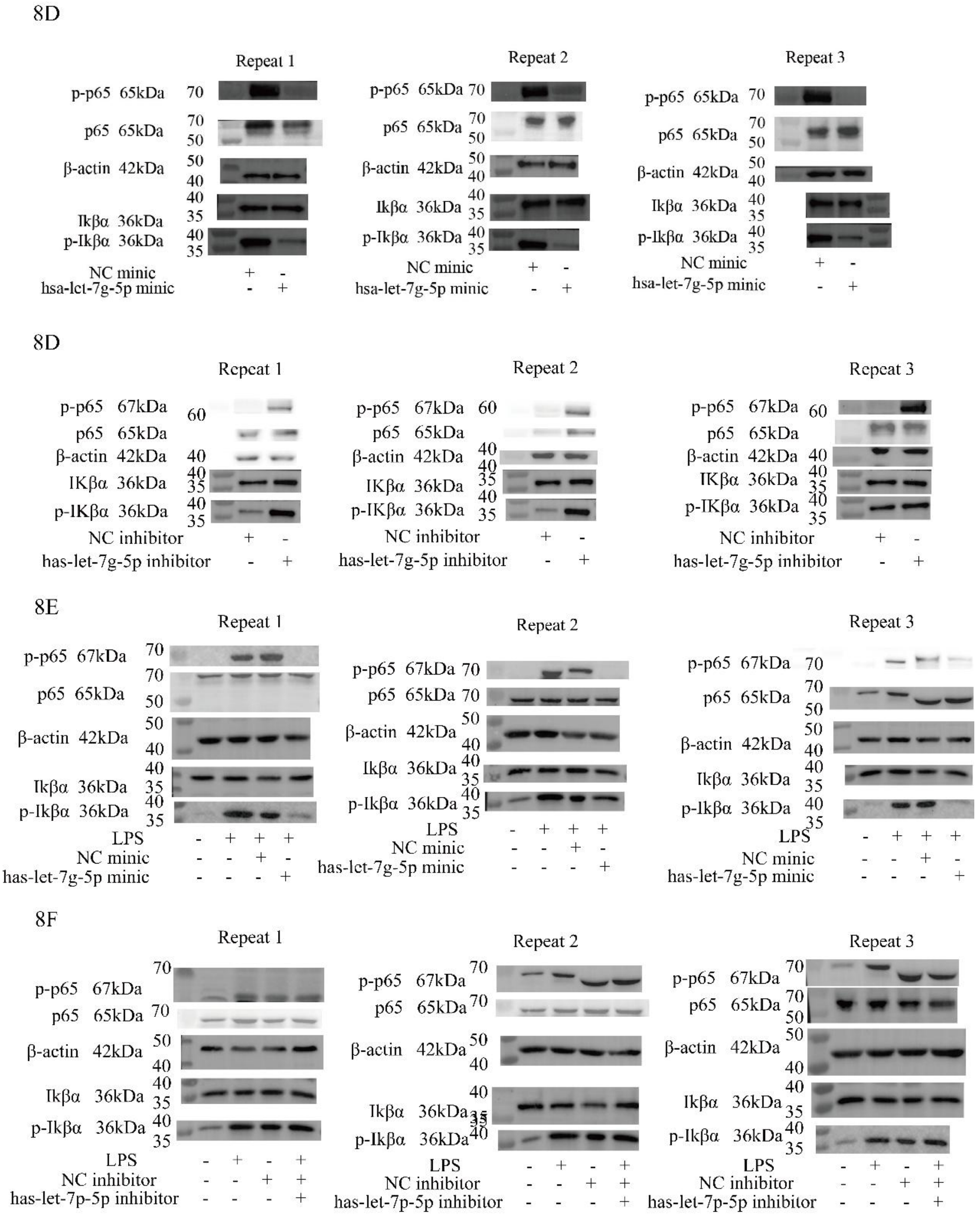

Supplementary Figure 8. Example of original western blot for three repeats (for Figure 8).
